# Spatially Resolved Transcriptomics Deconvolutes Prognostic Histological Subgroups in Patients with Colorectal Cancer and Synchronous Liver Metastases

**DOI:** 10.1158/0008-5472.CAN-22-2794

**Published:** 2023-04-14

**Authors:** Colin S. Wood, Kathryn A.F. Pennel, Holly Leslie, Assya Legrini, Andrew J. Cameron, Lydia Melissourgou-Syka, Jean A. Quinn, Hester C. van Wyk, Jennifer Hay, Antonia K. Roseweir, Colin Nixon, Campbell S.D. Roxburgh, Donald C. McMillan, Andrew V. Biankin, Owen J. Sansom, Paul G. Horgan, Joanne Edwards, Colin W. Steele, Nigel B. Jamieson

**Affiliations:** 1 University Department of Surgery, Glasgow Royal Infirmary, Glasgow, United Kingdom.; 2 School of Cancer Sciences, University of Glasgow, Glasgow, United Kingdom.; 3 Glasgow Tissue Research Facility, Queen Elizabeth University Hospital, Glasgow, United Kingdom.; 4 CRUK Beatson Institute, Glasgow, United Kingdom.

## Abstract

Strong immune responses in primary colorectal cancer correspond with better patient survival following surgery compared with tumors with predominantly stromal microenvironments. However, biomarkers to identify patients with colorectal cancer liver metastases (CRLM) with good prognosis following surgery for oligometastatic disease remain elusive. The aim of this study was to determine the practical application of a simple histological assessment of immune cell infiltration and stromal content in predicting outcome following synchronous resection of primary colorectal cancer and CRLM and to interrogate the underlying functional biology that drives disease progression. Samples from patients undergoing synchronous resection of primary colorectal cancer and CRLM were evaluated in detail through histological assessment, panel genomic and bulk transcriptomic assessment, IHC, and GeoMx spatial transcriptomics (ST) analysis. High immune infiltration of metastases was associated with improved cancer-specific survival. Bulk transcriptomic analysis was confounded by stromal content, but ST demonstrated that the invasive edge of the metastases of long-term survivors was characterized by adaptive immune cell populations enriched for type II IFN signaling and MHC-class II antigen presentation. In contrast, patients with poor prognosis demonstrated increased abundance of regulatory T cells and neutrophils with enrichment of Notch and TGFβ signaling pathways at the metastatic tumor center. In summary, histological assessment can stratify outcomes in patients undergoing synchronous resection of CRLM, suggesting that it has potential as a prognostic biomarker. Furthermore, ST analysis has revealed significant intratumoral and interlesional heterogeneity and identified the underlying transcriptomic programs driving each phenotype.

**Significance:**

Spatial transcriptomics uncovers heterogeneity between patients, between matched lesions in the same patient, and within individual lesions and identifies drivers of metastatic progression in colorectal cancer with reactive and suppressed immune microenvironments.

## Introduction

Colorectal cancer liver metastases (CRLM) remain the largest contributor to death for patients with colorectal cancer, with approximately 50% experiencing disease recurrence at this site (1). Modern surgical techniques, including portal vein embolization and parenchyma sparing surgery, have increased the number of patients eligible for resection; however, recurrence post-metastastectomy approaches 70% and intuitively, patients who recur have poorer outcomes (2, 3). To better predict outcome following CRLM resection, Fong and colleagues (4) derived a score based on clinicopathological factors, including nodal burden, synchronicity of presentation, number of CRLM and CEA level. More recently, genomic profiling has identified *KRAS*, *BRAF*, and *KRAS*/*TP53* co-mutations associating with poor prognosis (5–7); however, patient responses to treatment are unpredictable and successful development of novel therapies targeting stage IV colorectal cancer remains challenging (8). Expanding therapeutic targeting beyond mutational status to immune landscape and transcriptomic phenotypes may hold insight into the future management of CRLM (9).

Assessment of the tumor microenvironment (TME) in primary colorectal cancer, most notably the Immunoscore to determine the CD8/CD3^+^ T-cell ratio, has become a validated prognostic tool (10, 11). Simpler, yet powerful metrics evaluating hematoxylin and eosin (H&E)–stained sections include Klintrup–Mäkinen (KM) grade, assessing immune cell density at tumor invasive edge (IE) and tumor stroma percentage (TSP) measuring stromal density. Both have been applied to primary colorectal cancer; however, their prognostic utility in the metastatic setting remains unexplored, with biological interrogation also lacking (12, 13).

Four consensus molecular subtypes of primary colorectal cancer have been defined through bulk transcriptomic profiling generating a roadmap for translational colorectal cancer biology (14) but have failed to project into the metastatic setting (15). Increasingly, constraints of bulk transcriptomics are apparent with “stromal noise” potentially concealing biological pathway discovery and intratumoral heterogeneity characterization (16, 17). Although single-cell RNA sequencing (RNA-seq) has led to important discoveries (18, 19), crucially, topographic cellular orientation is lost along with vital biological insights relating to tissue morphology, cellular interactions and TME location-specific cellular expression. To overcome these limitations, spatial transcriptomics (ST) solutions aim to provide rich molecular insight while maintaining histological architecture. The Nanostring GeoMx Digital Spatial Profiler (DSP) is a novel ST platform enabling hi-plex, high-throughput characterization of user defined regions on formalin-fixed paraffin-embedded (FFPE) tissue (20) using UV-photocleavable barcodes hybridized to multiplex immunofluorescence (mIF)–stained tissue enabling up to whole-transcriptome analysis.

To better understand the mechanisms underlying metastatic development, the current study sought to investigate the prognostic utility of basic immunological assessments (KM and TSP) in a cohort of synchronously resected primary colorectal cancer with matched CRLM, integrating detailed IHC, genome panel, and bulk transcriptomic data to clearly define patient groups. Furthermore, an exploratory ST assessment using the Nanostring GeoMx DSP platform to interrogate the functional biology underlying clinically relevant subtypes in matched primary and CRLM to identify the features of disease progression was performed.

## Materials and Methods

### Cohort characteristics

Forty-one patients undergoing synchronous resection of primary colorectal cancer and CRLM with curative intent between April 2002 and June 2010 at Glasgow Royal Infirmary by a single surgeon (P.G. Horgan) were included (Table 1). Patients were identified from a prospectively maintained database and represent a consecutive cohort of resected patients with mature 10-year postoperative follow-up, including recurrence and mortality data. Patients were excluded if pathology slides were unavailable or if survival data were incomplete. Application to access patient tissue was authorized by the NHS Greater Glasgow and Clyde Biorepository under their NHS Research Ethics Committee approval with ethical approval granted in biorepository application #357, West of Scotland Ethics 22/WS/0207 in accordance with recognized ethical guidelines as described in the Declaration of Helsinki. Patients were followed up in the postoperative period at 1 month, then 6-monthly until 2 years, and thereafter annually until 5 years, at which time point they were discharged from follow-up. Date and cause of death was confirmed via access to the NHS Greater Glasgow and Clyde Clinical Portal. Records were complete until November 19, 2020, which served as the censor date. Cancer-specific survival was measured from the date of surgery until the date of death from colorectal cancer.

**Table 1.** Clinicopathological, morphological, and treatment characteristics for synchronously resected primary colorectal cancer and paired CRLM.

		Cohort *n* = 41 (%)	Median survival (mo)	Univariate HR (95% CI)	Multivariate HR (95% CI)
Age (<65)	<65	17 (41.5)	45	—	—
	>65	24 (58.5)	32	1.13 (0.55–2.32; *P* = 0.736)	—
Gender	Male	20 (48.8)	30	—	—
	Female	21 (51.2)	43	0.91 (0.45–1.84; *P* = 0.786)	—
ASA	I	4 (9.8)	38	—	—
	II	27 (65.9)	43	1.86 (0.43–7.94; *P* = 0.403)	—
	III	10 (24.4)	28	1.84 (0.39–8.69; *P* = 0.441)	—
Site	Colon	22 (53.7)	32	—	—
	Rectum	19 (46.3)	43	0.91 (0.45–1.86; *P* = 0.806)	—
T stage	T1/T2	3 (7.3)	27	—	—
	T3/T4	38 (92.7)	38	0.75 (0.18–3.17; *P* = 0.692)	—
N Stage	N0	17 (41.5)	62	—	—
	N1/N2	24 (58.5)	25	**2.54 (1.18–5.47**; ***P* = 0.017)**	2.57 (0.85–7.80; *P* = 0.095)
Number of metastases	1	24 (58.5)	30	—	—
	2–3	12 (29.3)	37	1.00 (0.44–2.24; *P* = 0.991)	—
	>3	5 (12.2)	47	1.20 (0.44–3.29; *P* = 0.718)	—
Size of metastases (cm)	<5	36 (87.8)	40	—	—
	>5	5 (12.2)	27	2.05 (0.76–5.53; *P* = 0.155)	—
R0 status (primary)	R0	29 (70.7)	30	—	—
	R1/R2	12 (29.3)	41	0.75 (0.34–1.64; *P* = 0.470)	—
R0 status (metastasis)	R0	28 (68.3)	46	—	—
	R1/R2	13 (31.7)	24	1.94 (0.93–4.04; *P* = 0.076)	—
Vascular Invasion (primary)	No	8 (19.5)	52	—	—
	Yes	33 (80.5)	34	1.13 (0.46–2.75; *P* = 0.793)	—
Fong Score	Low (1/2)	24 (58.5)	44	—	—
	High (3/4)	17 (41.5)	24	**2.25 (1.11–4.58**; ***P* = 0.025)**	1.16 (0.42–3.19; *P* = 0.778)
Klintrup Makinen Grade (primary)	Low	21 (51.2)	27	—	—
	High	20 (48.8)	60	**0.38 (0.17–0.86**; ***P* = 0.020)**	—
Klintrup Makinen Grade (metastasis)	Low	23 (56.1)	27	—	—
	High	18 (43.9)	57	**0.40 (0.19–0.86**; ***P* = 0.020)**	**0.36 (0.16–0.78**; ***P* = 0.010)**
TSP (primary)	Low^a^	18 (50.0)	36	—	—
	High	18 (50.0)	41	1.05 (0.49–2.29; *P* = 0.892)	—
TSP (metastasis)	Low^b^	18 (52.9)	32	—	—
	High	16 (47.1)	44	1.27 (0.57–2.86; *P* = 0.559)	—
Neoadjuvant treatment	No	29 (70.7)	27	—	—
	Yes	12 (29.3)	50	0.65 (0.29–1.46; *P* = 0.300)	—
Adjuvant chemotherapy	No	24 (58.5)	38	—	—
	Yes	17 (41.5)	34	1.61 (0.78–3.33; *P* = 0.196)	—

Note: Clinicopathological and treatment data for 41 patients managed by synchronous resection of primary colorectal cancer and CRLM are displayed. Univariate and multivariate survival analysis is presented and calculated using adjusted Cox proportional hazards regression model.

Abbreviations: ASA, American Society of Anesthesiologists Physical Status Classification; CI, confidence interval; HR, hazard ratio; TSP, tumor stromal percentage.

^a^36 primary colorectal cancer assessed for TSP.

^b^34 CRLM assessed for TSP.

### Immune landscape morphology analysis

Synchronously resected colorectal cancer and CRLM FFPE tissues were sectioned, stained with H&E, and scored using previously described methods (Supplementary Fig. S1; refs. 12, 21). KM grading of immune cell infiltration was scored from 0 to 3 for the depth of immune cells at the tumor invasive edge (IE) according to appearances at the deepest area of tumor invasion. Score 0, no increase in inflammatory cells at the deepest point of invasive margin; 1, mild and patchy increase in inflammatory cells; 2, a prominent inflammatory reaction forming a band at the invasive margin with some evidence of destruction of cancer cell islands; 3, a florid cup-like inflammatory infiltrate at the IE with frequent destruction of cancer cells. Tumors scored 2–3 were assigned KM^high^ with the remainder classified as KM^low^. For TSP evaluation, tumors with >50% stroma were classified as stroma^high^, with the remaining allocated as stroma^low^ (13). 15 primary colorectal cancer and CRLM were co-scored and in keeping with previously reported low interobserver variability (21), the average correlation of both scoring systems was 0.87 (high) between observers.

### GPOL panel mutational analysis

DNA was extracted from FFPE sections and standardized to a concentration of 4 ng/μL using the DNeasy kit (Qiagen). Mutational landscaping was performed by the Glasgow Precision Oncology Laboratory using an in-house genomic panel assay of 151 cancer-associated genes (Supplementary Table S1; ref. 22). Targeted capture libraries were prepared from 150 to 200-ng DNA. Sequencing was performed using an Illumina HiSeq400. The maftools (23) package was used to generate oncoplots, forest plots, and co-barplots to compare the mutational landscape for primary colorectal cancer and CRLM with morphological and immune cell integration.

### IHC

Detailed staining methods for CD3 and CD66b are included in Supplementary Methods and representative stained images with selected regions for analysis are demonstrated in Supplementary Fig. S2A–S2H. Visualization used the SlidePath digital image hub (Leica Biosystems) using a Hamamatsu NanoZoomer. QuPath was used for image analysis (Version: 0.3.2, University of Edinburgh, UK; ref. 24). From each primary and CRLM, 4 rectangular regions (mean perimeter 4,740-μm, mean area 1.38 mm^2^) corresponding to the IE and tumor center (TC) were annotated and positive cell detection was performed using in-built QuPath functionality. An RStudio pipeline was constructed (v1.2.1335 R Studio) to compare the number of positive cell detections per mm2 with the median cell count used to determine high and low cutoff value for categorical variables.

### Nanostring nCounter PanCancer IO360 bulk transcriptomic panel

Macrodissection of tumor regions, including TME and epithelium from unstained 10-μm-thick sections, was guided by an H&E image (see Supplementary Methods). RNA was extracted using the AllPrep DNA/RNA FFPE Kit (Qiagen) according to the manufacturer's protocol, using xylene for deparaffinization (average RNA concentration = 35 ng/μL). RNA quantity was assessed using RNA BR assay and the Qubit 2.0 fluorometer (Invitrogen, Life Technologies). RNA integrity (RIN) values were determined using Agilent 2100 Bioanalyzer (Agilent Technologies; maximum RIN = 2.1). Gene expression analysis was performed using the Nanostring nCounter IO360 panel (770 genes). Data acquisition was performed by using Nanostring's Digital Analyzer (FOV, 555). Raw gene expression count data were normalized using NanoString nSolver 4.0 software using 6 positive controls and 8 negative controls to account for background noise and sample variation across runs (GeNorm Algorithm).

### Nanostring GeoMx digital spatial profiling

The DSP protocol has been described by Merritt and colleagues (20) and consists of slide preparation, including antigen retrieval and staining with immunofluorescent markers, hybridization of tissue with UV-photocleavable probes, scanning, region selection, probe collection, library preparation and sequencing. Supplementary Methods provide detailed description of slide preparation. Four channels are available for the detection of four customizable morphology markers: FITC/525nm, Cy3/568nm, Texas Red/615nm, and Cy5/666 nm (20). One channel is reserved for the nuclear stain (DAPI). In this experiment, Pan-Cytokeratin (PanCK), CD45 for global immune cell population and αSMA for fibroblasts and collagenous architecture constituted the other channels. The primary colorectal cancer and paired CRLMs were mounted on the same slide (4 matched pairs on 4 slides) before hybridization with the Cancer Transcriptome Atlas (CTA) panel of probes corresponding to 1,825 genes (Nanostring). The CTA panel is designed to comprehensively characterize immune activity and tumor biology within the TME (25).

Following hybridization, the slides were scanned on the GeoMx instrument with the workspace used to select regions of interest (ROI). In CRLM, distinct regions at the IE were observed, those that stained strongly for CD45 were annotated as “metastatic Invasive Edge—immune” (mIE; pink). ROIs at the IE of metastases that stained more prominently for αSMA were annotated as “metastatic Invasive Edge—stroma” (mSE; yellow). PanCK-stained epithelial regions in the center of CRLM were annotated as “metastatic Tumor Center” (mTC; blue). In primary colorectal cancer, central epithelial regions were annotated as “primary Tumor Center” (pTC; dark green) and regions at the IE where there was a clear interface between epithelium and TME comprising clear immune cell populations were annotated as “primary Invasive Edge” (pIE; light green). 3 of the primary lesions had tertiary lymphoid regions (pTLR; brown). One TLR was identified within metastasis (mTLR; orange). Within each primary and CRLM, ROIs were selected with a range of mTC, mIE, mSE, and mTLR (Supplementary Figs. S3–S6). Each of the 48 circular ROIs were 500-μm diameter (area 196,795 μm^2^) with average nuclei count of 1,371 (range, 707–2,055).

Once ROI selection was complete, collection was initiated whereby photo-cleavable oligonucleotide probes in the ROIs were exposed to UV-light to cleave the UV-sensitive probes. The released probe-specific DSP barcodes were then aspirated from selected ROIs and collected into the 96-well DSP plate. The probes, following rehydration with DEPC-treated water, were then added to the corresponding well of a new 96-well PCR plate containing the GeoMx Seq Code primers and the PCR Master Mix. Details of library preparation and sequencing are included in Supplementary Methods.

### Survival analysis

All analyses using RStudio used v1.2.1335 of RStudio (R build version 4.1.1). Kaplan–Meier survival analysis curves (log-rank test) were generated using *survival* (26) and *survminer* (27) packages. Cox-regression analysis was performed using *finalfit* (28) and *hmisc* (29) packages. Statistical significance was set to a *P* value of <0.05 unless otherwise stated.

### IO360 panel and GeoMx ST analysis

IO360 panel analysis data underwent QC and normalization in the proprietary nCounter Advanced Analysis Suite. For the GeoMx data, the Digital Count Conversion files were uploaded onto the GeoMx DSP analysis suite (Nanostring), where they underwent quality control (QC), filtering, Q3 normalization, and background correction. The normalized counts from each were downloaded into RStudio. Principal component analysis (PCA) was performed using *prcomp* (base R) and plotted using *ggplot2* (30). Differential Gene Expression (DGE) was performed using the exact test as part of edgeR package (31). Volcano plots were generated using *ggplot2* (30). Heatmaps were generated using *ComplexHeatMap* (32). Gene set enrichment analysis (GSEA) was performed using the *fgsea* package (33). Single-Sample GSEA (ssGSEA) was performed using the *GSVA* package (34). *ClusterProfiler* (35) was used to interrogate the Reactome-curated database (36). Immune cell spatial deconvolution for nCounter IO360 data was performed in the nCounter Advanced Analysis suite with RStudio used for analysis and visualization. Immune spatial deconvolution of GeoMx-derived CTA data was performed using the Bioconductor *SpatialDecon* tool (37).

### Data availability

Data generated using the Nanostring GeoMx DSP platform are available from Zenodo at https://doi.org/10.5281/zenodo.7520788. Data generated using the Nanostring nCounter platform are available from GEO under accession number GSE224235 (https://www.ncbi.nlm.nih.gov/geo/query/acc.cgi?acc=GSE224235). Any other data generated in this study are available upon request from the corresponding author.

## Results

### Clinicopathological and morphological characteristics determine patient outcome

Baseline clinicopathological and treatment details for the 41 patients with synchronously resected colorectal cancer and CRLM are described in Table 1. 59% were ≥65-years-old with similar gender distribution. Both rectal (46%) and colonic (54%) primary colorectal cancers were included, with the majority being stage T3 or T4 (93%), 59% had lymph node metastases and 29% received neoadjuvant chemotherapy. The 5 and 10-year mortality rate was 64% and 82%, respectively.

The impact of traditional clinicopathological variables on outcome was assessed (Table 1). N-Stage (HR, 2.54; *P* = 0.017) and Fong Score (HR, 2.25; *P* = 0.025) were prognostic on univariate analysis. Gross morphological immune and stromal feature evaluation demonstrated KM^high^ in the primary colorectal cancer (HR, 0.38; *P* = 0.02) and CRLM (HR, 0.40; *P* = 0.02) predicted cancer-specific survival (Table 1). In a multivariate model, KM^high^ within CRLM was the most significant prognostic factor (HR, 0.36; *P* = 0.01).

### Genomic characterization of synchronous CRLM

**Figure 1.**
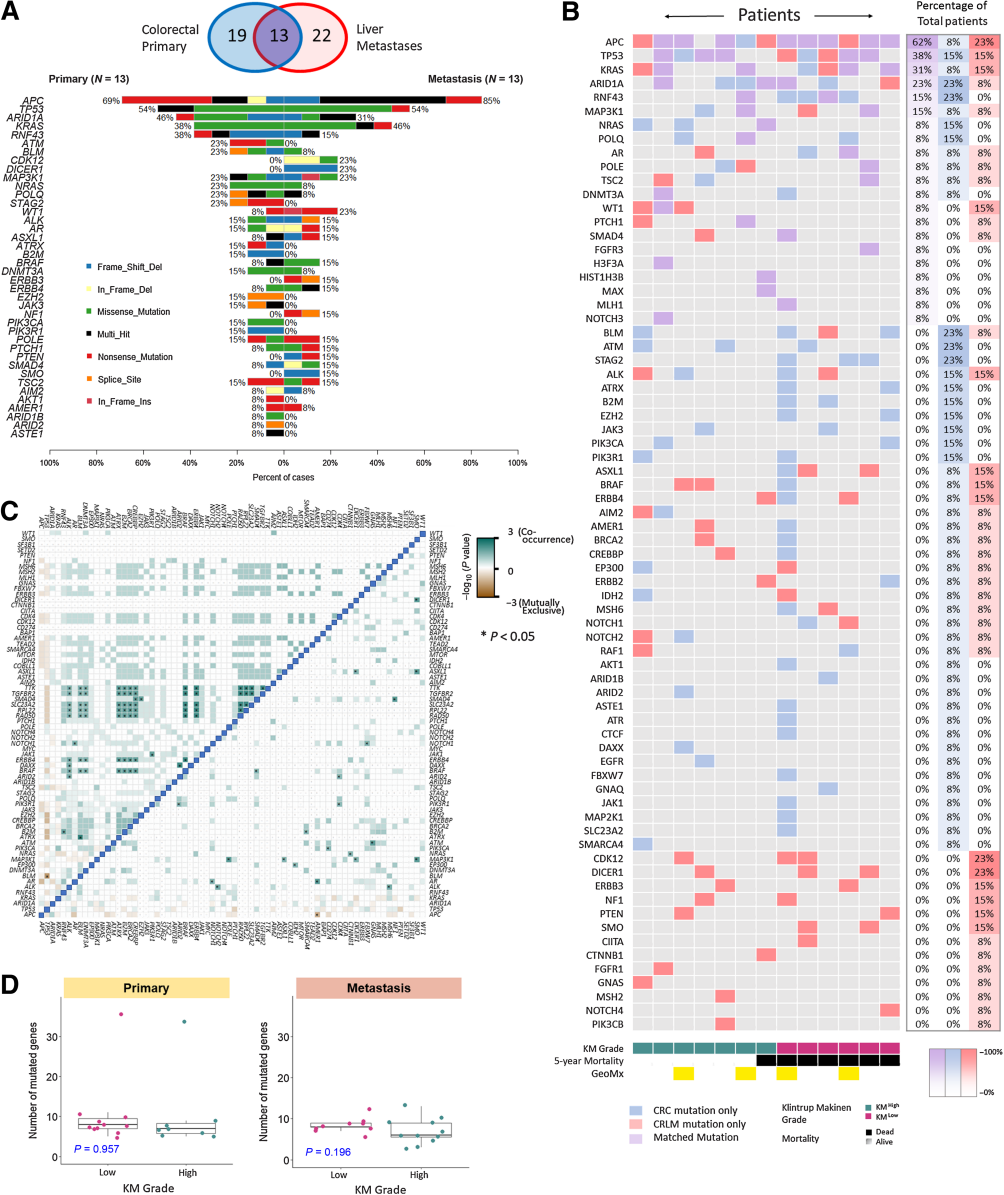
Mutational characterization of primary colorectal cancer and CRLM. **A,** Venn diagram demonstrating primary colorectal cancer and CRLM that underwent genomic analysis. Co-Barplot illustrating most frequently mutated genes across 13 matched primary colorectal cancer and CRLM, including mutation type. Genes are ordered by mutational frequency. Sections were sequenced using GPOL (Glasgow Precision Oncology Laboratory) mutational panel. **B,** Oncoplot demonstrating concurrent mutations in the 13 matched lesions. Patients are ranked according to co-mutational burden on the *y*-axis and ranked according to KM grade on the *x*-axis. Blue, gene mutated in primary only; red, gene mutated in metastasis only; purple, mutated in primary and metastasis. The right-hand three columns denote the percentage of total patients with each mutation type. **C,** Correlation matrix demonstrating co-occurrence of mutations, with left of the blue demarcation line representing primary colorectal cancer, right of the blue demarcation line representing CRLM (pair-wise Fisher exact test; *, *P* < 0.05). Gene names are displayed along the *x*- and *y*-axes ordered by mutational frequency. Dark green boxes represent significant co-occurrence. **D,** Box plot illustrating mutational burden in primary colorectal cancer and CRLM according to KM grade using the Mann–Whitney test to assess for statistically significant difference between KM groups.

Mutational analysis was performed to determine whether genomic landscape underpins immune morphology in colorectal cancer and paired CRLM. Gene mutation panel profiling for 19 primary colorectal cancers and 22 CRLMs was performed, of which, 13 were matched pairs (Fig. 1A). For the most frequently mutated genes, a similar mutation rate was noted between primary colorectal cancer and CRLM (Fig. 1A): *APC* 69% primary colorectal cancer and 85% CRLM; *TP53* 54% and 54%; *ARID1A* 46% and 31% and *KRAS* 38% and 46%. These genes were also the most frequently co-mutated genes between paired sites (Fig. 1B). Out-with the frequently occurring mutations, rarer mutations were less likely to be concordant between sites. There were no significantly discordant genes between primary colorectal cancer and CRLM (Supplementary Fig. S7A); however, *CDK12*, *ERBB3* and *DICER1* were exclusively mutated in CRLM (Supplementary Fig. S7A). *APC*, *TP53*, *ARID1A*, and *KRAS* mutations were mutually exclusive of other mutations in both primary colorectal cancer and CRLM (Fig. 1C). Multiple clusters of co-occurring mutations were noted, including *BRAF*, *ERBB4*, *BRCA2*, and *TGFBR2*, which associated with two patients with hypermutation status (>10 mutations). These were present in primary colorectal cancer, only suggesting the hyper-mutated state was not conserved between primary and CRLM.

Although we expected tumor mutational burden to associate with immune morphology, neither KM grade nor TSP were associated with mutational frequency or landscape (Fig. 1D). *KRAS*, *TP53* co-mutation was more common in CRLM (6) than colorectal cancer (3). Of the patients with *KRAS*, *TP53* CRLM co-mutation, 5 died early following surgery (range, 20–42 months) whereas only one of these patients survived long term (Log-rank, *P* = 0.26, Supplementary Fig. S7B). No other genomic subgroups associated with prognosis.

### Bulk transcriptomic analysis of prognostic subtypes

**Figure 2.**
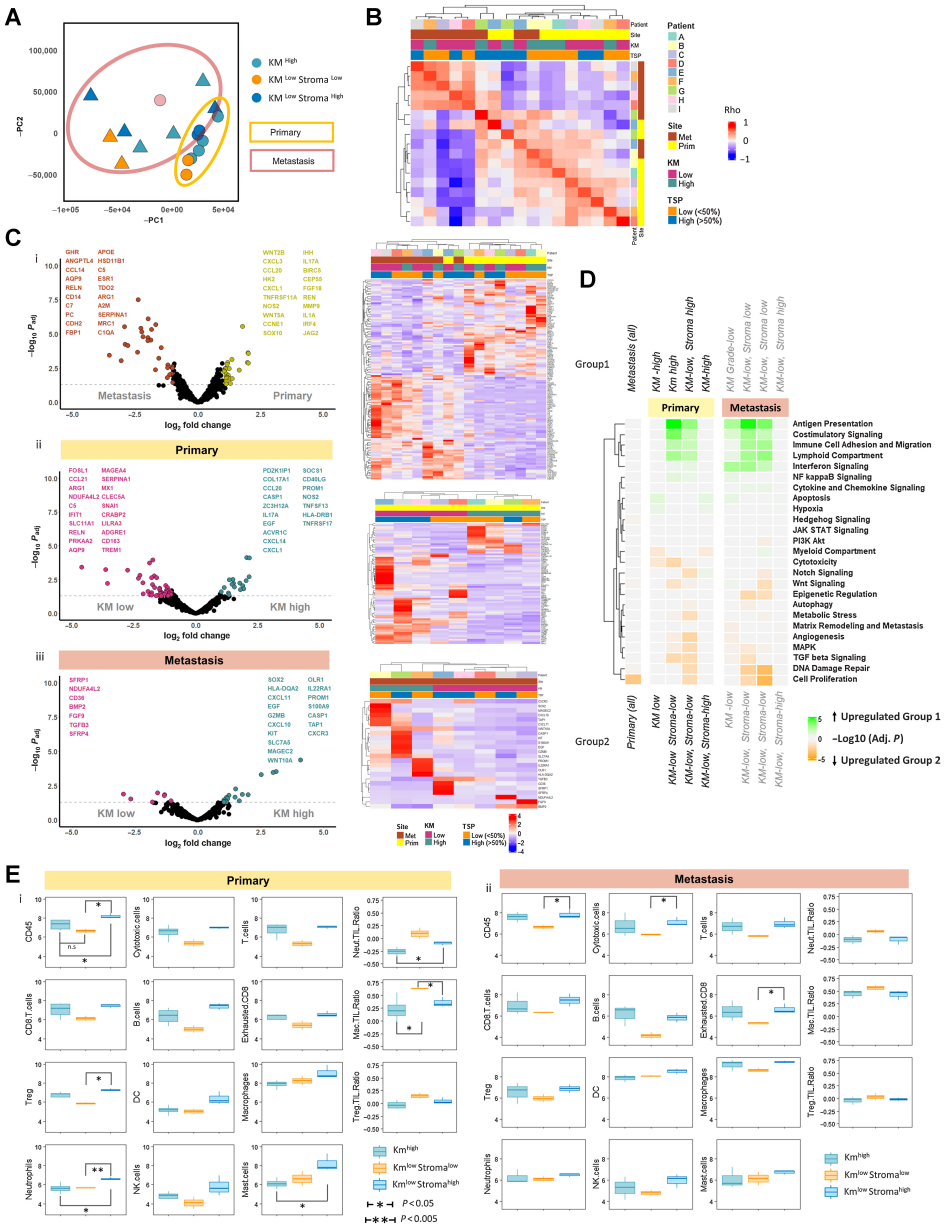
BULK IO360 transcriptomic characterization of matched primary colorectal cancer and CRLM. **A,** PCA plot demonstrating two principal components of minimal variance for all samples. Primary colorectal cancer samples are demonstrated by circles and yellow outline. CRLM are demonstrated by triangular points and brown outline. KM^high^, KM^low^ Stroma^low^ and KM^low^ Stroma^high^ samples are depicted by color. **B,** Unsupervised analysis using gene expression correlation matrix for all samples. Patient, site, KM grade, and TSP are depicted by key. Spearman correlation of all expressed genes performed between each sample sequenced and plotted on the heatmap. *k*-means clustering of heatmap to demonstrate correlated samples. Red, strong correlation. Blue, negative correlation. **C,** Volcano plots demonstrating differential gene expression results and clustered heatmap of significant genes for (i) all primary colorectal cancer versus CRLM; (ii) KM grade: KM ^high^ versus KM ^low^ primary colorectal cancer; (iii) KM grade: KM ^high^ versus KM ^low^ CRLM. The *x*-axis of volcano plot demonstrates log_2_-fold change; *y*-axis demonstrates –log_10_*P*. Colored points demonstrate significant changes in gene expression between groups (*P* < 0.05 and logFC > 1.5). Volcano plots demonstrate top 20 differentially expressed genes for each group. **D,** Heatmap demonstrating GSEA results comparing the different tumors grouped according with KM grade and TSP using io360-curated gene sets annotated on the right of the diagram. Heatmap squares represent log_10_-adjusted *P* value. Green, upregulation in group 1; orange, upregulation in group 2. The heatmap is clustered by *y*-axis only to demonstrate frequently upregulated gene sets. **E,** Box plot comparisons of immune cell populations and selected cell:cell ratios between KM grade and TSP segregated groups using deconvolution software included in the nCounter package. Annotated subgroups are: Km^high^, Km^low^ Stroma^low^, Km^low^ Stroma^high^. The *y*-axis represents log_10_ of estimated cell count. Primary colorectal cancer is represented in i and CRLM in ii.

To gain insight into the gene expression profile associated with immune morphology subtypes, 9 matched primary colorectal cancer and CRLM were selected for nCounter bulk transcriptomic IO360 panel analysis (Supplementary Fig. S8). PCA plot (Fig. 2A) demonstrates clear separation of primary colorectal cancer (*N* = 9) and CRLM (*N* = 8) with wide interlesional discrimination according to KM and TSP status evident for both primary and metastases (one CRLM failed QC).

An unsupervised hierarchical clustering correlation matrix confirmed that tumor site was the predominant determinant of transcriptomic profile (Fig. 2B). When segregated into primary colorectal cancer and CRLM groups, KM status further discriminates gene expression at both sites, particularly within CRLM (Supplementary Fig. S9A and S9B). These gene lists were defined by DGE and predictably the highest number of differentially expressed genes resulted from primary colorectal cancer and CRLM comparison (Fig. 2C). Between DGE of KM^high^ and KM^low^ in primary and CRLM, there was minimal overlap. An apparent stromal subgroup was apparent within KM^low^ lesions (Fig. 2C).

GSEA comparing primary colorectal cancer and CRLM demonstrated a single significantly upregulated pathway in primary colorectal cancer [cell proliferation, normalized enrichment score (NES) = 2.05; *P*_adj_ = 0.002; Fig. 2D]. KM^low^ lesions were separated into KM^low^ Stroma^low^ and KM^low^ Stroma^high^ groups according to the PCA plot (Fig. 2A). When pairwise comparison of each group was performed using GSEA and plotted in a clustered heatmap, concordant immune pathways enrichment for KM^high^ and KM^low^ Stroma^high^ tumors was demonstrated in both primary colorectal cancer and CRLM, including antigen presentation, costimulatory signaling, immune cell adhesion and migration, lymphoid compartment, and IFN signaling (Fig. 2D). These data suggest that despite significant prognostic and histological differences, KM^high^ and KM^low^ Stroma^high^ lesions demonstrate similar bulk transcriptomic immune pathway dysregulation. In contrast, KM^low^ Stroma^low^ lesions were characterized by aberrations of cell proliferation, DNA damage repair, and TGFβ signaling at both primary and metastatic site.

Immune cell deconvolution of the IO360 panel transcriptome data demonstrated that although T and B cells’ abundance was similar for KM^high^ and KM^low^ Stroma^high^ lesions across primary colorectal cancer and CRLM, in KM^low^ Stroma^low^ lesions these populations were less prevalent (Fig. 2E). Differences were less notable in myeloid-derived cell populations (macrophages, neutrophils, and NK cells). To highlight these differences in cell populations, immune cell ratios were calculated demonstrating that neutrophil: T-cell, macrophage: T-cell and NK: T-cell ratios were highest in KM^low^ Stroma^low^ lesions, most significantly in primary colorectal cancer (Fig. 2E). These data may suggest high tumor stroma content disproportionately confounding the bulk transcriptomic signal.

### Immune cell characterization defines KM phenotype

**Figure 3.**
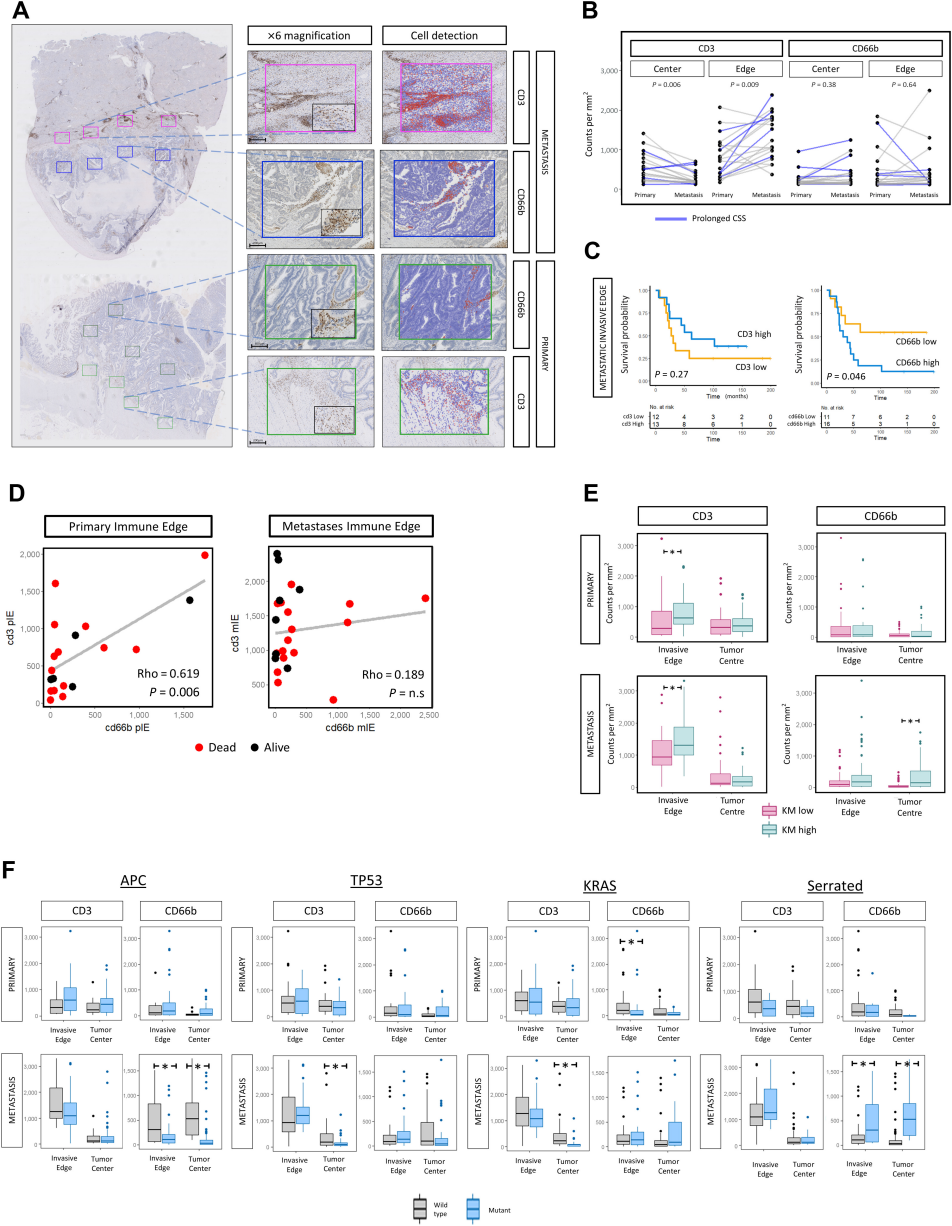
IHC characterization of matched primary colorectal cancer and CRLM with integration of morphological and mutational features. **A,** Representative images of CD3 and CD66b immunohistochemical staining (Patient B). Whole section demonstrated at ×0.5 magnifications; ROIs corresponding to tumor center (TC) and invasive edge (IE) of primary and CRLM are shown at ×6 and ×10 (black box). Scale bar, 100 μm. **B,** Intrapatient comparison between primary and metastasis of CD3 and CD66b cell counts at tumor center and invasive edge. *P* values calculated using the Mann–Whitney test. **C,** Kaplan–Meier survival plots (log-rank test, *P* values displayed) demonstrating the prognostic impact of CD3 and CD66b cell density at CRLM IE identified by IHC for IE of CRLM. High and low values determined according to median expression. **D,** Comparison of CD3 and CD66b cell density at (i) primary IE, (ii) CRLM IE, and (iii) CD3 primary IE and metastatic TC. Spearman Rho analysis. **E,** Box plots illustrating the relationship between KM grade and CD3 and CD66b cell counts at the IE and TC of primary colorectal cancer and CRLM. The *P* value was calculated using the Mann–Whitney test. **F,** Box plot representing relationship between mutational features (APC, TP53, KRAS, Serrated) and CD3 and CD66b cell density at TC and IE of primary colorectal cancer and CRLM. The *P* value was calculated using the Mann–Whitney test. Lesions of serrated origin were defined as APC^wild^ + KRAS/BRAF^mutation^.

IHC was performed to further delineate the role of specific immune cells in prognostic subtypes. Samples were stained for CD3 (T-lymphocytes; primary *n* = 21; CRLM *n* = 28) and CD66b (granulocytes/neutrophils; primary *n* = 21; CRLM *n* = 29) based on tumorigenic role of neutrophils demonstrated in a preclinical model by our group (38). Representative images are shown in Fig. 3A. Increased CD3 density was demonstrated at the CRLM IE compared with primary colorectal cancer IE [median 683.5, interquartile range (IQR): 272–1,039 vs. median 1,142.2, IQR: 810–1,702, *P* = 0.009; Fig. 3B)]. Conversely, CD3 density was reduced in the CRLM TC compared with primary colorectal cancer TC (median 190, IQR: 71–273 vs. median 443, IQR: 150–629, *P* = 0.006; Fig. 3B). CD66b count in contrast did not differ significantly between primary and metastasis at TC or IE (Fig. 3B).

Survival analysis was performed according to CD3 and CD66b expression across all lesions and regions. CD66b^high^ at the IE of CRLM was associated with worse prognosis (5-year survival 19% vs. 64%, Log-rank, *P* = 0.046; Fig. 3C).

Co-abundance analysis of CD3 and CD66b demonstrated that within primary colorectal cancer IE, CD3 and CD66b were strongly correlated (rho = 0.619, *P* = 0.006); however, this was not the case in the CRLM (rho = 0.189, *P* = n.s.; Fig. 3D).

CD3 and CD66b cell density data were then integrated with immune morphological features. CD3 density was elevated at the IE for both colorectal cancer (*P* = 0.02) and CRLM (*P* < 0.005; Fig. 3E) in the KM^high^-grade tumors. CD66b density was elevated in the TC of KM^high^ CRLM (*P* < 0.005), suggesting that a primed immune response was concordant with infiltration of neutrophils to the CRLM center. Immune cell abundance and distribution was then assessed according to mutational status, demonstrating that *KRAS* mutation was associated with reduced CD3 density in the TC of CRLM (*P* < 0.005; Fig. 3F), suggesting adaptive immune exclusion. APC mutation associated with reduced CD66b density in the TC and IE of CRLM (*P* < 0.005). We categorized tumors with *APC* wild-type and *BRAF* or *KRAS* mutation as serrated, a subtype of colorectal cancer that derive from serrated polyps via an alternate pathway usually with absence of APC mutation. We found evidence of elevated CD66b density in the IE and TC of CRLM (*P* < 0.005), suggesting predisposition to neutrophil infiltration in metastases of serrated origin (Fig. 3F).

### Spatially resolved transcriptomic analysis demonstrates marked intratumoral and intertumoral heterogeneity and biological insights into prognostic subtypes of CRLM

**Figure 4.**
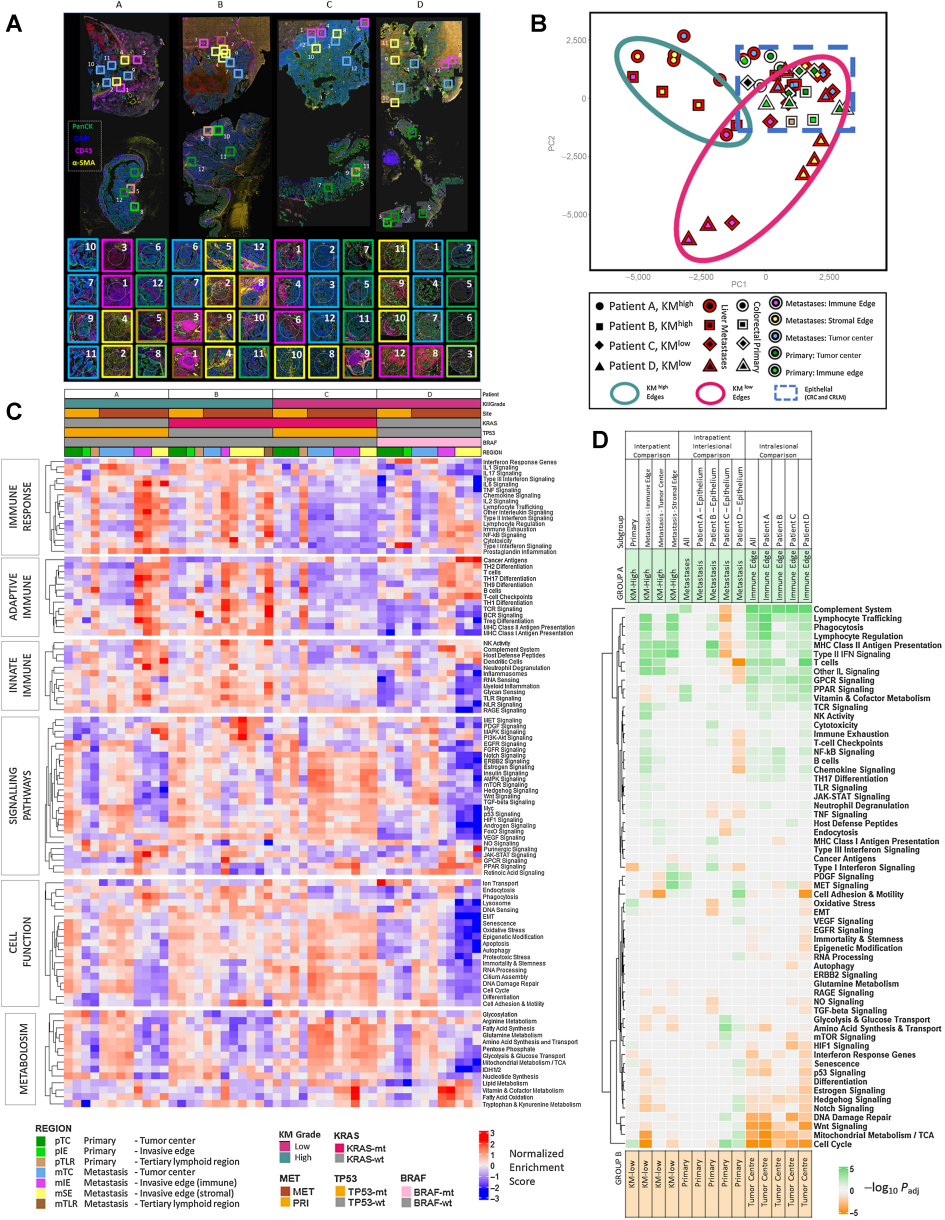
Spatially resolved transcriptomic analysis using Nanostring Cancer Transcriptome Atlas gene sets. **A,** Representative images of mIF staining of four matched primary colorectal cancer and CRLM. DAPI, blue; Pancytokeratin, green; CD45, pink; αSMA, yellow. Topographic regions are annotated and each box represents hand-selected area of tumor. Eight regions were taken from CRLM and four from primary colorectal cancer per patient. Patient A: KM^high^, KRAS-wt, good prognosis. Patient B: KM^high^, KRAS-mt, good prognosis. Patient C: KM^low^, KRAS/TP53 co-mutation, poor prognosis. Patient D: KM-low, KRAS-wt, BRAF-mt high-mutational burden lesion. **B,** PCA plot of all ROIs selected. The patient from whom the lesion originated is represented by shape. A red border indicates region arises from CRLM and white border represents primary colorectal cancer. The topographical region within the lesion is illustrated by the innermost color of the shape. KM ^high^ metastatic edges, green circle; KM^low^ metastatic edges, red circle; dashed blue line, epithelial regions of primary colorectal cancer and CRLM. **C,** Heatmap demonstrating single sample GSEA for every ROI, ordered on the *x*-axis by patient and ROI. Key presented to aid patient identification. The *y*-axis represents annotated gene sets from Cancer Transcriptome Atlas ordered by and clustered within modules of Immune Response, Adaptive Immune, Innate Immune, Signaling Pathways. Cell Function, Metabolism. Each cell represents the NES scaled by pathway. **D,** Heatmap demonstrating GSEA providing interpatient comparison of selected areas between KM^high^ and KM^low^ patients and intrapatient comparison between primary and metastatic sites and intralesional comparison between tumor center and immune edge. Subset of regions filtered before GSEA is demonstrated in subgroup. Subsequent groups compared in GSEA identified as groups A and B. Group A annotated at top of diagram and represented by green. Group B annotated at bottom of diagram and represented by orange. Cells of heatmap represent −log_10_(*P*_adj_) for comparison; cell is tinted green if pathway is upregulated in group A and orange if upregulated in group B.

Four matched primary colorectal cancer and CRLM were selected for ST analysis, the mIF-stained images and ROI are demonstrated in Fig. 4A. Two of the matched samples were KM^high^ (Patients A and B) and two were KM^low^ (Patients C and D) at primary and metastatic sites, and all samples were Stroma^low^. The IE of both KM^high^ CRLMs were characterized by an αSMA–stained sIE region encapsulating the PanCK-stained mTC with abundant CD45^+^ cells with numerous TLRs between the border of the sIE capsule and normal liver parenchyma (Supplementary Figs. S3 and S4). Both KM^high^ CRLM also demonstrate tumor necrosis centrally. In contrast, both KM^low^ CRLM had a sharper transition between epithelial mTC and normal liver parenchyma with limited αSMA and CD45 staining, suggesting a less well-defined IE (Supplementary Figs. S5 and S6).

The IO360 bulk transcriptome panel and GeoMx CTA data in matched samples were correlated and grouped by region (Supplementary Fig. S10). Gene expression for all GeoMx regions correlated well with the corresponding matched bulk sample (rho, 0.612–0.906, all *P* < 0.005); however, ROIs from primary colorectal cancer and the mIE of CRLM correlated most strongly.

A PCA plot of all ROIs demonstrated that most heterogeneity exists in the invasive edges (mIE, sIE) of CRLM, whereas epithelial regions, including TC of primary colorectal cancer (pTC) and CRLM (mTC) clustered closely (blue box, Fig. 4B). The IEs of KM^high^ and KM^low^ tumors clustered separately for CRLM. However, the mIE from patient C (*KRAS/TP53* co-mutation), clustered more closely with the epithelial group.

ssGSEA was performed across all ROIs using CTA-derived curated gene modules (Immune Response, Adaptive Immune, Innate Immune, Cancer Signaling, Cell Function and Metabolism) demonstrating biological differences both between similarly annotated regions in different patients and between different regions within the same lesion (Fig. 4C). To corroborate these observations, DGE and GSEA were performed using interpatient, intrapatient and intralesional subgroup comparisons (Fig. 4D). For validation, REACTOME-curated gene sets were applied with similar results obtained (Supplementary Fig. S11; Supplementary Table S3).

### Intrapatient, interlesional heterogeneity demonstrates relative immunosuppression in CRLM in KM ^low^ patients

Epithelial regions from primary colorectal cancer and CRLM within the same patients were compared. Although there were no significantly dysregulated gene pathways between primary and CRLM for patient A (KM^high^), in patient B (KM^high^) upregulation of immune signaling in the CRLM included MHC class II antigen presentation (NES = −2.29; *P*_adj_ <0.005) and type II IFN signaling (NES = −2.05; *P*_adj_ < 0.005; Fig. 4D). In KM^low^ patients (Patients C and D), there was relative downregulation of immune related pathways in the CRLM, including lymphocyte trafficking (NES = 1.91; *P*_adj_ < 0.005) and type II IFN signaling (NES = 2.11; *P*_adj_ < 0.005), chemokine signaling (NES = 1.76; *P*_adj_ < 0.005), and T-cell checkpoints (NES = 1.78; *P*_adj_ = 0.01; Fig. 4D).

### Good prognosis high-immune subgroups are defined transcriptomically by immune signaling pathways at metastatic invasive edges

The KM^high^ CRLMs (Patient A and B) demonstrated enrichment of adaptive immune and immune response CTA gene set modules most profoundly at the mIE with the following altered: B-cell signaling (NES = −1.9; *P*_adj_*<* 0.005); T-cell signaling (NES = −2.05; *P*_adj_*<* 0.005); MHC class II antigen presentation (NES = −2.09; *P*_adj_ < 0.005); type II IFN signaling (NES = −2.05; *P*_adj_ < 0.005); lymphocyte regulation (NES = −1.81; *P*_adj_ < 0.005); lymphocyte trafficking (NES = −2.06; *P*_adj_ < 0.005; Fig. 4D). In contrast, patient C (KM^low^*KRAS*/*TP53* co-mutation) was immune deplete across all regions of the CRLM according to transcriptomic assessment (Fig. 4C). Although immune signaling was ubiquitous across most immune pathways in the mIE of KM^high^ lesions, in Patient D (KM^low^, *BRAF* mutation) there was reduced expression of type II and III IFN signaling and MHC class I and II antigen presentation (Fig. 4C); however, with high complement system expression at the mIE (NES = −2.68; *P*_adj_ < 0.005). The KM^low^ CRLMs demonstrate enrichment of atypical immune pathways according to ssGSEA analysis with Patient C having upregulation of RAGE signaling (Innate Immune gene module) with cancer antigens and TH9 differentiation (adaptive Immune gene module) upregulated in Patient D (Fig. 4C).

### Poor prognostic KRAS, TP53 co-mutated patient demonstrates upregulation of NOTCH and TGFB signaling in the metastatic tumor center

Gene sets within the cancer signaling pathways module were then analyzed identifying 3 clusters (Fig. 4C). In the first cluster, MAPK, MET, PDGF and PI3K expression appear enriched at the IE of KM^high^ lesions with MET (NES = −2.27; *P*_adj_ < 0.005) and PDGF (NES = −2.14; *P*_adj_ = 0.01) most significantly expressed at the mSE (Fig. 4D). The second cluster was enriched for VEGF, WNT, NOTCH, and Hedgehog signaling throughout regions of Patient C and the mTC of Patient D. Comparing the mTC of KM^high^ and KM^low^ lesions, NOTCH signaling (NES = 1.72; *P*_adj_ = 0.022) and TGF-B signaling (NES = 1.70; *P*_adj_ = 0.027) were most significantly upregulated in low immune lesions (Fig. 4D). The third cluster included Purinergic and JAK–STAT gene sets that were most highly expressed in mIE and mSE of *KRAS* wild-type lesions (Fig. 4C).

### KM^low^ CRLM show greater mitotic and metabolic activity than KM^high^ lesions

Next, pathways associated with cellular function and metabolism were analyzed demonstrating that KM^low^ lesions, particularly Patient C (*KRAS/TP53* co-mutation), were mitotically and metabolically active, particularly at the mTC (Fig. 4C). Comparing the mTC of KM^high^ and KM^low^ patients, cell adhesion and motility was significantly upregulated in KM^low^ CRLM (NES = 2.11; *P*_adj_ < 0.005). At the mIE, cell cycle (NES = 2.21; *P*_adj_ < 0.005) and mitochondrial metabolism (NES = 2.36; *P*_adj_ < 0.005) were upregulated in KM^low^ lesions (Fig. 4D).

### Expression of bulk transcriptomic signatures in ST datasets

The expression of established bulk transcriptomic signatures in this ST dataset was explored. To date the most comprehensive characterization of CRLM using bulk transcriptomic techniques was performed by Pitroda and colleagues (15) who described 3 CRLM mRNA signatures that were integrated with clinicopathological traits and classified as; 1, canonical; 2, immune; and 3, stromal. In this dataset, the genes from all 3 signatures that overlapped with the CTA-panel were markedly overexpressed in the IE of CRLM compared with the TC further demonstrating the capability of ST to demonstrate intratumoral heterogeneity and the potential stromal contamination of bulk signatures (Supplementary Fig. S12A and S12B).

### Spatial deconvolution of tumor regions demonstrates distinctive regional immune cell populations between KM and mutational subgroups

**Figure 5.**
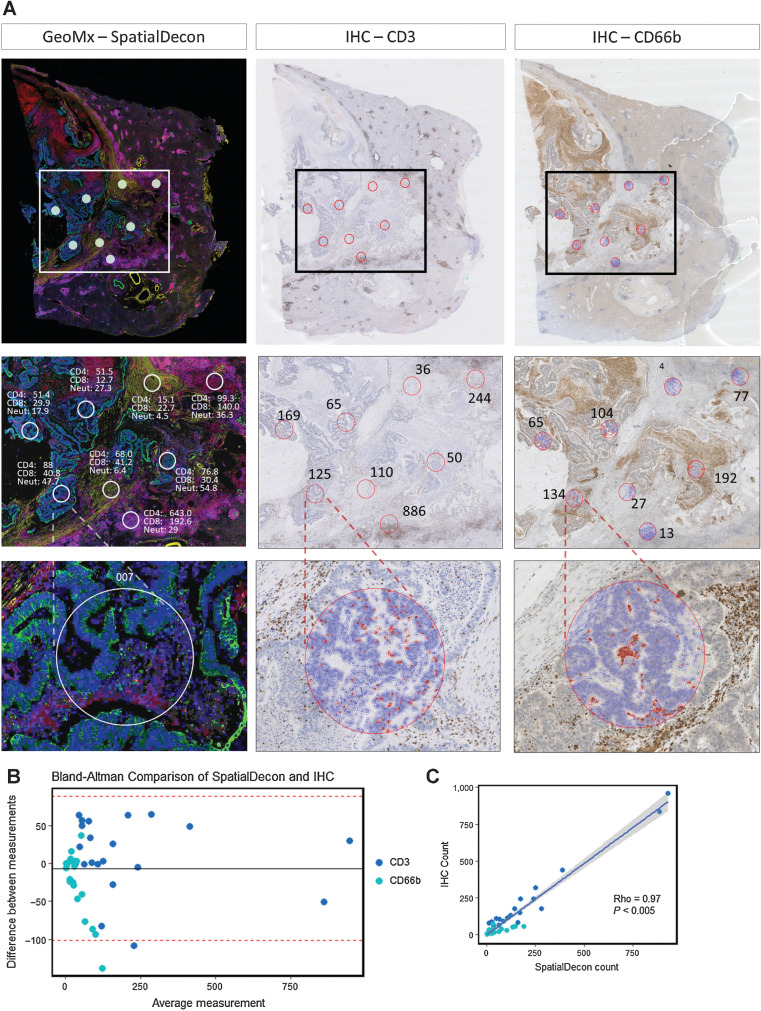
Immune cell spatial deconvolution. **A,** Representative images from 1 CRLM (Patient A, Fig. 4A) showing cell detection from the Qupath package used on a CD3 and CD66b IHC-stained liver metastasis to count number of CD3 and CD66b-positive cells from 21 regions from a total of three CRLM. This count was compared with the SpatialDecon-derived count that uses the transcriptomic data from the corresponding ROI in the GeoMx mIF-stained matched sample (See Supplementary Fig. S5). **B,** Bland Altman plot comparing transcriptome SpatialDecon-derived cell count versus the IHC-derived cell count. **C,** Correlation plot comparing transcriptome SpatialDecon-derived cell count versus the IHC-derived cell count.

**Figure 6.**
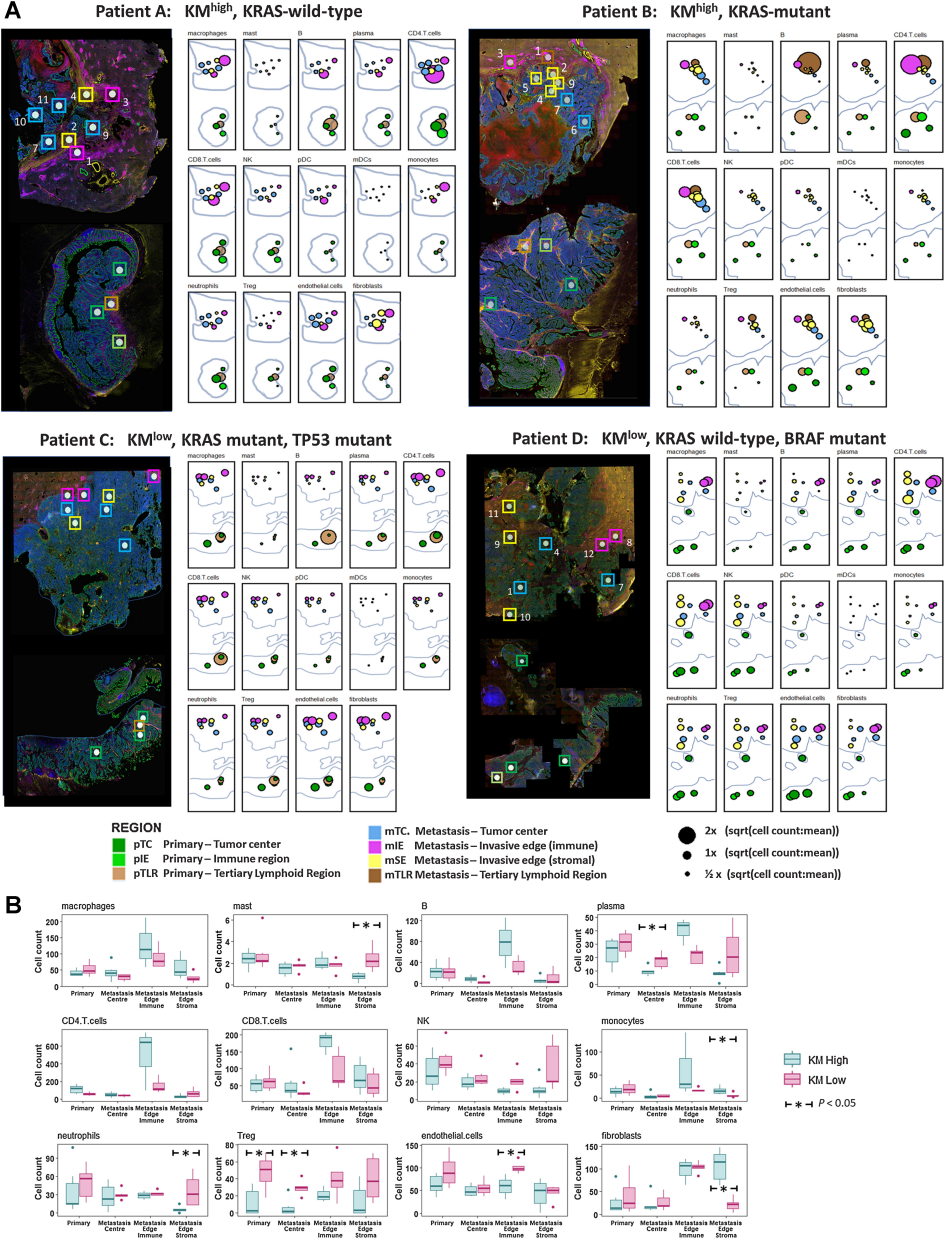
Topographic immune cell deconvolution primary colorectal cancer and CRLM. **A,** Images (from Fig. 4A) of primary colorectal cancer (bottom) and CRLM (top) with 48 ROIs superimposed. Abundance estimates as determined from transcriptome by SpatialDecon for 14 cell populations illustrated for each ROI with color coding detailing the annotated tumor region. Radius is proportional to the estimated cell counts within the ROI. The immune cell count per region was extracted and the square root of the ratio to the mean immune cell count per region (41.37) of all immune cells was calculated and is displayed. The square root of the ratio was calculated to minimize the skew caused by variance of highly expressed cell types. **B,** Box plots demonstrating the median and interquartile range for each cell type analyzed organized by cell type and topographic region and grouped by KM grade. All ROIs taken from primary colorectal cancer except TLR are grouped as primary. Dendritic cells were removed due to insignificant counts. The Mann–Whitney test was used to assess for statistical significance; *, *P* < 0.05.

To further interrogate the topographic cellular differences between clinically relevant subgroups, spatial deconvolution was performed to estimate relative immune cell abundances within ROIs using the SpatialDecon tool (37). Selected validation of the transcriptomic deconvolution analysis was achieved by comparison with chromogenic IHC density data from serial tissue sections (Fig. 5A). For CD3 and CD66b, we demonstrated that ST immune cell abundance matched closely the IHC cell count (Bland Altman plot, Fig. 5B; rho = 0.97 *P* < 0.005, Fig. 5C). We subsequently illustrated that immune cell populations have distinct spatial distributions with marked immune cell heterogeneity noted between matched primary colorectal cancer and CRLM, tumor ROIs and immune subgroups (Fig. 6A).

The mIE demonstrated higher abundance of adaptive immune cells compared with mTC (CD4: mean 280.0 vs. 46.8, *P* < 0.005; CD8: mean 126.8 vs. 43.4, *P* < 0.005; B cells: mean 48.5 vs. 5.8, *P* < 0.005; Fig. 6B; Supplementary Table S4). According to KM status, KM^high^ lesions had higher CD4 (mean 498.5 vs. 150.3, *P* = 0.22) and B cells (mean 77.8 vs. 30.9, *P* = 0.23) but only CD8 (mean 180.0 vs. 93.5, *P* = 0.035) was significantly elevated compared with KM^low^ (Fig. 6B). Although regulatory T cells (Treg) density was uniform across topographic tumor regions, according to immunological subtype, KM^low^ tumors demonstrated elevated density compared with KM^high^ tumors (mean 12.2 vs. 40.0, *P* < 0.005) across all topographic regions (Fig. 6B).

Macrophages demonstrated a topographic distribution similar to CD4 and CD8 cells with higher abundance in mIE regions compared with mTC (mean 103.0 vs. 36.1, *P* = 0.008; Fig. 6B). However, macrophage density did not differ according to KM status across mIE regions (KM^high^ 127.8 vs. KM^low^ 88.1, *P* = 0.47). Neutrophils and natural killer (NK) cells demonstrated similar relative topographic patterns, with uniform abundance across ROIs. There were significantly higher levels in KM^low^ tumors (neutrophils: mean 37.8 vs. 23.7, *P* < 0.005; NK cells: mean 33.1 vs. 19.5, *P* < 0.005) with the greatest difference observed in the mSE (neutrophils: mean 36.9 vs. 5.74, *P* = 0.05; NK cells: mean 37.0 vs. 12.7, *P* = 0.12; Supplementary Table S4). The invasive edges of KM^high^ and KM^low^ CRLM also differed with regards to the fibroblast populations with the former characterized by high mSE fibroblast density (Mean 107.9 vs. 21.2, *P* < 0.005).

The small number of TLRs precluded statistical comparison; however, we noted that the dominant cell types in all TLRs were adaptive immune cells, particularly B cells, with comparatively higher abundance of macrophages, monocytes, and Treg cells in TLR in primary colorectal cancer of Patient C (Supplementary Fig. S13).

## Discussion

Interest in a personalized oncology approach to the management of colorectal cancer incorporating prognostic and predictive biomarkers continues to build; however, heterogeneity of the tumoral immune response may initiate tumor evolution and impede individualized management algorithms. The application of ST strategies to comprehensively atlas separate compartments, including epithelial and microenvironments, not only at multiple topographical regions but also temporally through analysis of metastases has potential to deepen our understanding of tumor immune interactions. Furthermore, it may result in an armamentarium of biomarkers to guide management and target therapeutics.

Our analysis of primary colorectal cancer and synchronous CRLM has revealed that KM^High^ grade defines a group of patients, regardless of genomic background, with a favorable prognosis following resection of advanced metastatic disease. We therefore demonstrate that in addition to density, location of immune cells is critical to outcome prediction in these patients. The tumor immune geography is not accurately captured by approaches that fail to incorporate the histology or topographic regions of the tumor, including flow cytometry, bulk RNA-seq, and scRNA-seq. We have demonstrated the fidelity of ST analysis in this context, enabling accurate and discriminating characterization of the transcriptome of invasive margins of these tumors both at the primary and secondary sites.

The varying response to surgical resection of CRLM has driven the need for impactful biomarkers. The Immunoscore is a validated immunological metric for primary nonmetastatic colorectal cancer; however, the utility in Stage IV disease has only recently been explored. In characterizing the immune “multiverse” within a matched primary colorectal cancer and CRLM cohort, Van den Eynde and colleagues (39) demonstrated comprehensively, as corroborated in this study, vast intra-metastatic, intersite, and interpatient heterogeneity and postulate this as a potential mechanism for treatment resistance (39). In characterizing the invasive margin and tumor center, the authors demonstrate topographic insights, with higher whole-slide immune infiltration in smaller metastases and dense hotspots in larger CRLM. Furthermore, it was noted that in patients with multiple metastases, the immune infiltration of the least infiltrated CRLMs determines outcome most accurately. Several adaptive immune cell markers were characterized and their findings of CD3 abundance in the CRLM invasive margin with pockets of CD20 cells distributed throughout CRLMs were corroborated within our current analysis. The findings reported here supplement this work by demonstrating that innate immune cell populations, particularly neutrophils at the invasive edge, may play a prominent immunosuppressive role in CRLM. The prognostic role of the Immunoscore obtained from the primary lesion in patients with metastatic colorectal cancer was not investigated in the work by Van den Eynde and colleagues (39); however, previous data suggest limited benefit (40). This contrasts with the KM score that we have demonstrated remains prognostic regardless of the evaluation within the primary or metastases. To increase the understanding of the tumor immune interface, this study focused spatially resolved transcriptional analysis at the pivotal tumor-invasive edge that the Immunoscore targets.

Two prognostic subtypes of CRLM have been identified according to broad morphological and biological characterization. Patients with CRLM experiencing a prolonged survival (KM^high^) following resection are characterized by a fibroblast-rich stromal capsule enriched for PDGF and MET signaling, surrounded by abundant T-lymphocytes with enrichment of MHC Class II Antigen Presentation and Type 2 IFN Signaling. In contrast, CRLM in patients with a poor prognosis (KM^low^) were characterized by epithelial regions infiltrated by Treg, poorly defined invasive edges infiltrated by neutrophils and sparse-adaptive immune cells in combination with overwhelming downregulation of adaptive immune signaling pathways. ST further demonstrated distinct differences in the metastatic immune edge of two patients with poor prognosis thus illustrating interlesional topographical heterogeneity. In one patient with *KRAS, TP53* co-mutation we observed a devoid immune landscape at the invasive edge. This patient, uniquely in this dataset, demonstrated upregulation of RAGE signaling in the CRLM. A recent *in vitro* cell-culture study demonstrated RAGE-mediated chemotaxis of immunosuppressive myeloid-derived suppressor cells that may offer one possible explanation for our findings in this CRLM (41). In contrast, a patient with hypermutated genome and BRAF mutation demonstrated downregulation of specific immune pathways, including Type-II IFN and MHC-Class II antigen presentation, highlighting their pivotal importance. Intriguingly, ST demonstrated expression at the invasive edge of this CRLM, upregulation of cancer/testis antigens, a group of antigens expressed only on germ cells or tumors (42), demonstrating the potential for novel discovery and potential patient-specific targets uncovered by an ST strategy.

The findings in this study corroborate previous definitions of three prognostic transcriptomic subtypes of CRLM, one of which was a good prognostic high-immune subtype with demonstrable IFN-related pathways (15). Through integrative genomic analysis, we have shown that a TP53/KRAS co-mutated lesion demonstrated profound adaptive immunosuppression, enrichment of NOTCH signaling and TGFβ signaling pathways in the epithelial center of metastases. This builds upon our recent discovery from a TP53/KRAS co-mutated genetically engineered mouse model (GEMM) in which epithelial NOTCH expression drove aggressive metastatic progression through TGFβ signaling and neutrophil recruitment (38). Interestingly, the analysis of immune cell populations suggests a propensity for neutrophil recruitment to BRAF/KRAS mutant CRLM in the absence of APC mutations, corroborating observations from serrated colorectal cancer murine models in human patients (38). Importantly, this GEMM was found to respond favorably to inhibition of TGFβ signaling pathways, limiting metastatic progression. This highlights the potential of an ST strategy to disentangle the complexity of TME, resulting in identification of clinically relevant tumor subgroups vulnerable to immune-focused therapeutic options in the future.

The current study suggests that in the metastatic context, mutational landscape appears to impact outcome less than the host immune response to the CRLM. Through transcriptomic immune cell deconvolution, a marked heterogeneity in immune cell populations was uncovered according to prognostic subgroups both temporally and spatially throughout a patient's burden of disease. For CRLM with transcriptomic evidence of immunosuppression, Treg cells appear paramount, as we observed a high density located in epithelial regions of primary and CRLM. Schürch and colleagues (43) recently used hi-plex CODEX deep-phenotyping to extensively map the immune landscape of primary colorectal cancer, highlighting similar importance of immunosuppressive Treg cells, particularly in cellular neighborhoods where antigen presentation occurs, with a negative effect on outcome.

In primary colorectal cancer, high stromal content may be indicative of an evolving metastatic process, underpinned by epithelial–mesenchymal transition, and associated with poor outcome (44, 45). However, in the current study, TSP assessment conferred no prognostic influence when present in the metastatic setting. An affiliate group recently demonstrated that extensive stroma in the primary tumor confounded bulk transcriptomic analysis approaches (17). They propose that transcriptomic data from targeted areas of the tumor may be an effective strategy to filter out stromal “noise” and more readily obtain pertinent biological data, overcoming the stromal effect also demonstrated in our data in bulk RNA-seq analysis.

### Limitations and future strategy

These data were generated from a single-center, single-surgeon experience in a predominantly treatment naïve cohort limited to synchronously resected colorectal cancer primary and CRLM. The power of this cohort is derived from linkage of the primary and secondary site of disease, clearly revealing the prognostic importance of the KM grade in this context. We acknowledge that these pilot data interrogated only a small selection of samples on the GeoMx platform; however, we believe that even in a limited sample size, this technology has demonstrated biological insight that we will now explore with greater power in larger cohorts of CRLM. Although the bespoke region selection offered by GeoMx offers flexibility and configurability, the possibility of region selection bias introduced by the user and subsequent lost regions of biological importance is a possibility. Alternative ST platforms exist, including the VISIUM (10X Genomics) platform that in contrast provides a more comprehensive topography of a single larger area (6.5 mm^2^) per section through sequencing of 5,000 barcoded dots (46). In this study, the CTA has offered insight into differences in immune and cancer signaling pathways between prognostic groups; however, employment of a whole-transcriptome pipeline may have facilitated a more powerful discovery approach. Finally, our region selection strategy did not use segmentation, and therefore each region had mixed epithelial and stromal components. Our future GeoMx experiments will take advantage of more advanced segmentation and cell detection strategies.

The novel technology demonstrated in this article uniquely characterizes the source tissue with multiplex immunofluorescent staining to produce high-resolution images while simultaneously producing high-plex, high-throughput transcriptomic data. Correlation of these 2 facets will unleash fascinating insights but will require advanced machine learning algorithms and artificial intelligence, which remain under development, combined with advanced image analysis techniques. Artificial intelligence is being incorporated into routine digital pathology and alongside development of these technologies will help personalize treatment decisions in future.

### Conclusion

In conclusion, the invasive edge of CRLM influences outcome in this resected cohort and can be thoroughly characterized using ST analysis, and in future may allow researchers to fully address the reason for resistance to therapies and recurrence following surgery. This ST study of a synchronously resected cohort of colorectal cancer and CRLM has demonstrated novel insights into the pathways driving tumor progression on an individual patient basis. This study illustrates the potential to leverage ST performed on the GeoMx DSP to characterize tumor heterogeneity, and identify novel biomarkers associated with clinically relevant subtypes of colorectal cancer. This is a highly utilizable platform in FFPE tissues from human and preclinical models. There was high concordance between spatial immune cell deconvolution with IHC protein assessment and future work will seek to integrate deep immuno-phenotyping, transcriptomic output and multiomic integration, with a view to applying this technology at scale in addition to serial analysis of lesion through interrogation of biopsy specimens.

## Supplementary Material

Supplementary Data All supplementary figures and supplementary tables 1 and 2 from the manuscript

Supplementry Table 3: REACTOME Gene Set Enrichment Analysis results Gene Set Enrichment Analysis results obtained by interrogating ranked list of differentially expressed KM high vs KM low genes against the REACTOME curated gene set database using ClusterProfiler package

Supplementary Table 4: SpatialDecon derived immune cell counts SpatialDecon derived immune cell counts aggregated by Region, KM grade and KRAS status with pairwise comparison between groups. Mann-Whitney test used to determine statistical significance between groups

Supplementary Methods Supplementary details regarding methods and materials
