# Supplementary material for: Spatially Resolved Transcriptomics Deconvolutes Prognostic Histological Subgroups in Patients with Colorectal Cancer and Synchronous Liver Metastases
Source: Cancer Res. 2023 Apr 14;83(8):1329–44. doi: 10.1158/0008-5472.CAN-22-2794 (PMC10102851; doi:10.1158/0008-5472.CAN-22-2794)
Supplement: Supplementary Data — All supplementary figures and supplementary tables 1 and 2 from the manuscript [file can-22-2794_supplementary_data_suppst1-st2sf1-sf13.pdf]

ABCB1	CSF1R	FGFR4	KMT2A	PHF6	SLC22A1	ZMYM3
ABCC2	CTNNB1	FLT1	KRAS	PIK3CA	SLC22A2	
ABL1	CYP19A1	FLT3	LAMA2	PIK3R1	SLC31A1	
ABL2	CYP2A6	FLT4	LCK	PSMB1	SLC34A2	
AKT1	CYP2B6	FSTL5	LTK	PSMB2	SLC45A3	
AKT2	CYP2C19	GNA11	MAP2K1	PSMB5	SLCO1B1	
AKT3	CYP2C9	GNAQ	MAP2K2	PSMD1	SMAD4	
ALK	CYP2D6	GNAS	MAP2K4	PSMD2	SMARCA4	
APC	DDR1	GSTP1	MAP3K1	PTCH1	SMARCB1	
ASXL1	DDR2	H3F3A	MAPK1	PTEN	SMO	
ATM	DDX3X	HNF1A	MED13	PTPN11	SNCAIP	
ATRX	DNMT3A	HRAS	MET	RAF1	SOS1	
BRAF	DPYD	IDH1	MLH1	RARA	SPRED1	
BRCA1	EGFR	IDH2	MPL	RARB	SRC	
BRCA2	ERBB2	IKZF1	MST1R	RARG	STK11	
CBL	ERBB3	IL2RA	MTOR	RB1	SUFU	
CDA	ERBB4	IL2RB	MYC	RET	TAS2R38	
CDH1	ERG	IL2RG	MYD88	ROS1	TET2	
CDKN2A	ESR1	INPP4B	NELL2	RPS6KB1	TP53	
CDKN2B	ESR2	JAK1	NF1	RUNX1	TRRAP	
CEBPA	EZH2	JAK2	NOTCH1	RXRA	TYK2	
CHD7	FBXW7	JAK3	NPM1	RXRB	UGT1A1	
CHIC2	FGFR1	KDM6A	NRAS	RXRG	VHL	
CREBBP	FGFR2	KDR	PDGFRA	SHH	WT1	
CRLF2	FGFR3	KIT	PDGFRB	SHOC2	YES1	

Supplemental Table 1:

GPOL 151 cancer-associated gene panel

Step	Temp.	Time	Cycles
UDG incubation	37°C	30 min	1 X
UDG deactivation	50°C	10 min	1 X
Initial denaturation	95°C	3 min	1 X
Denaturation	95°C	15 sec	
Anneal	65°C	60 sec	18 X
Extend	68°C	30 sec	
Final extension	68°C	5 min	1 X
Hold	4°C	∞	1 X

Supplemental Table 2:

The cycling conditions of the PCR plate during the library preparation. Protocol provided by Nanostring.

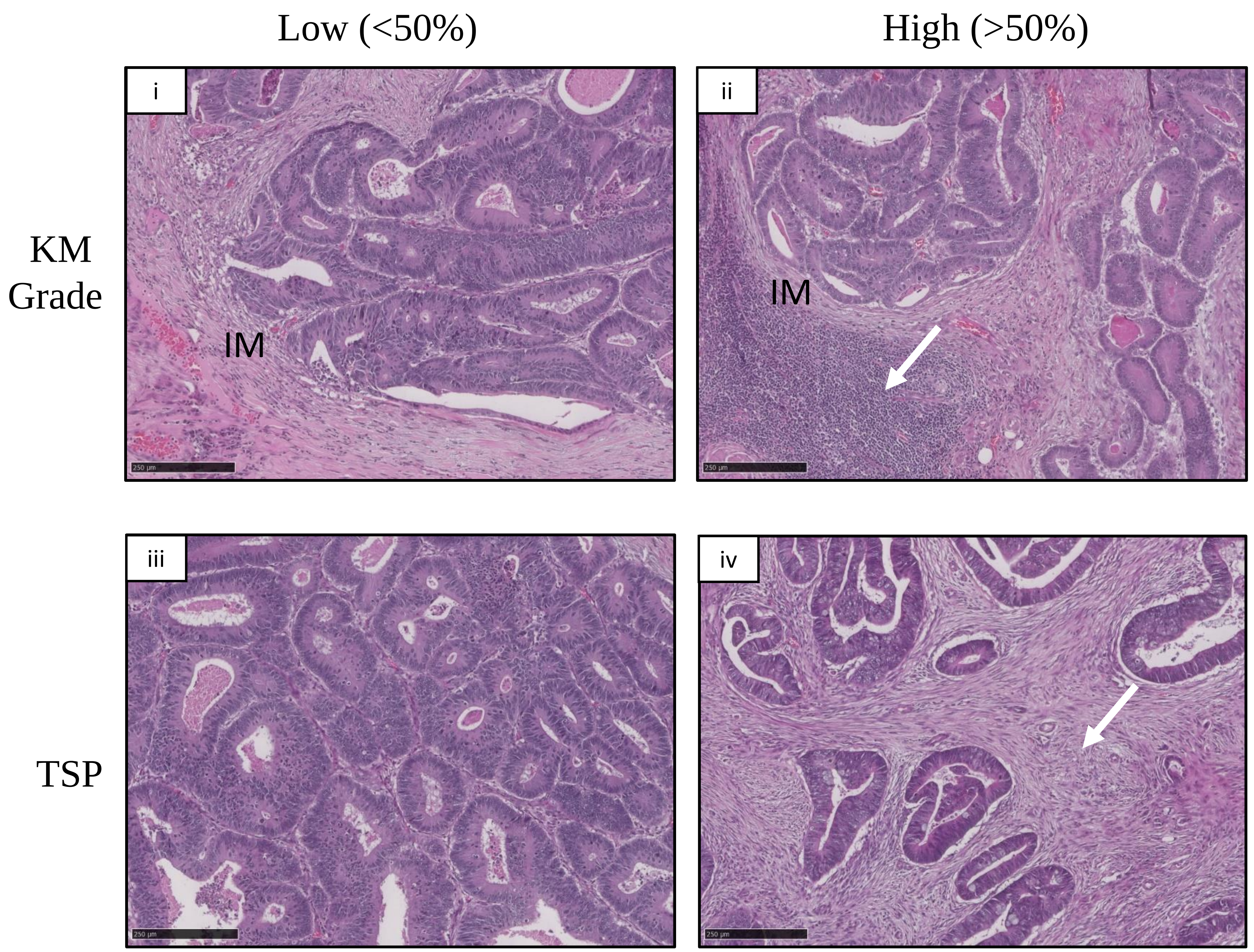

Supplementary Figure 1:
Representative images of H&E sections for scoring of Klintrup-makinen KM grade and Tumor stroma percentage) TSP of primary colorectal cancer.

Images taken at x10 magnification (NDP Viewer software), scale bar 250µM. (i) KM^{low} graded tumor (ii) KM^{high} tumor (high immune infiltration arrowed). (iii) low intra-tumor stromal percentage (TSP) (<50%) (iv) high (>50%) TSP tumor (stromal area arrowed) . IM: Invasive margin

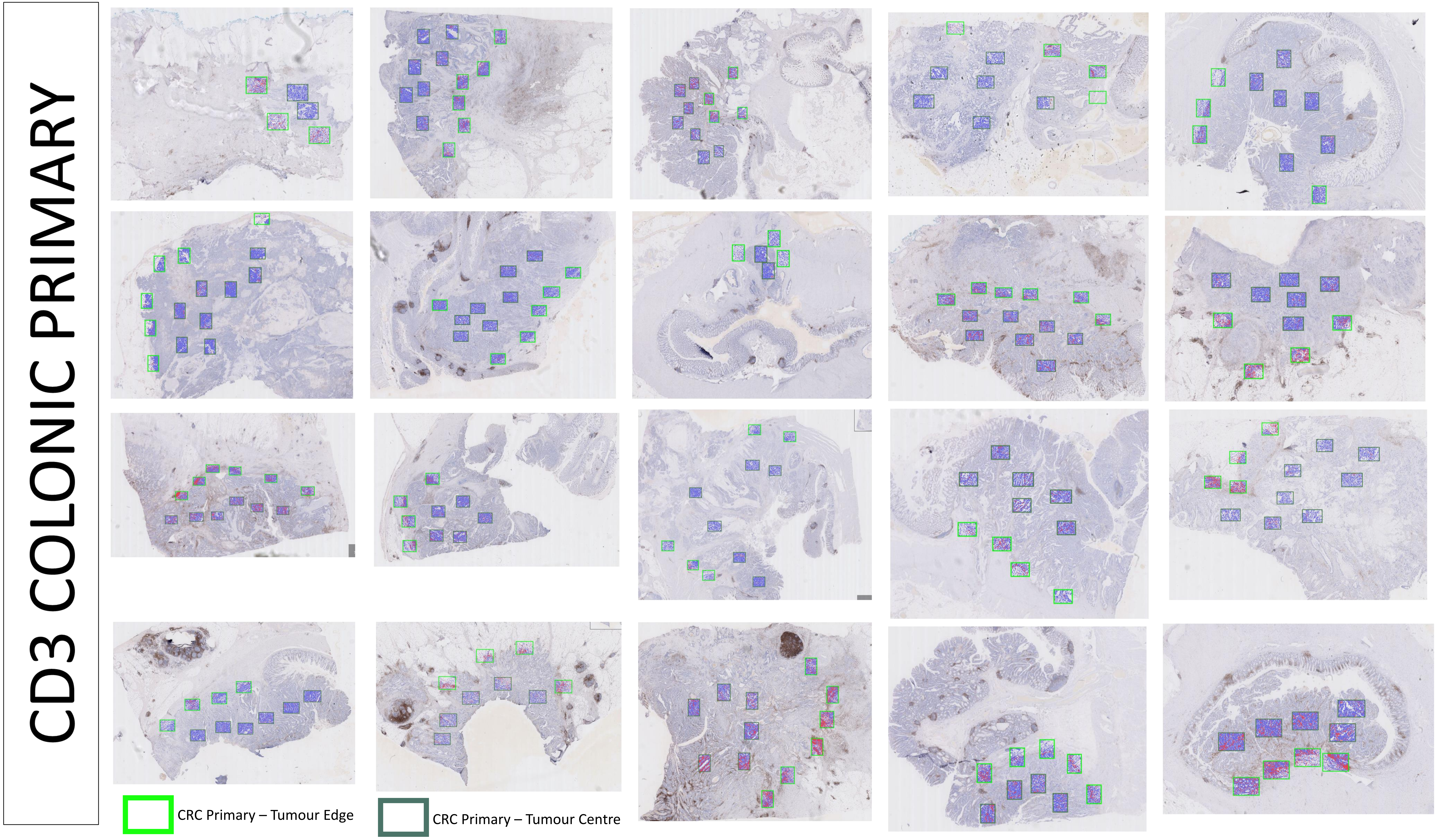

Supplementary Figure 2a:
Representative images of CD3 stained IHC of Colorectal Cancer Primary
CRC: Colorectal Cancer   CRLM: Colorectal Liver Metastasis

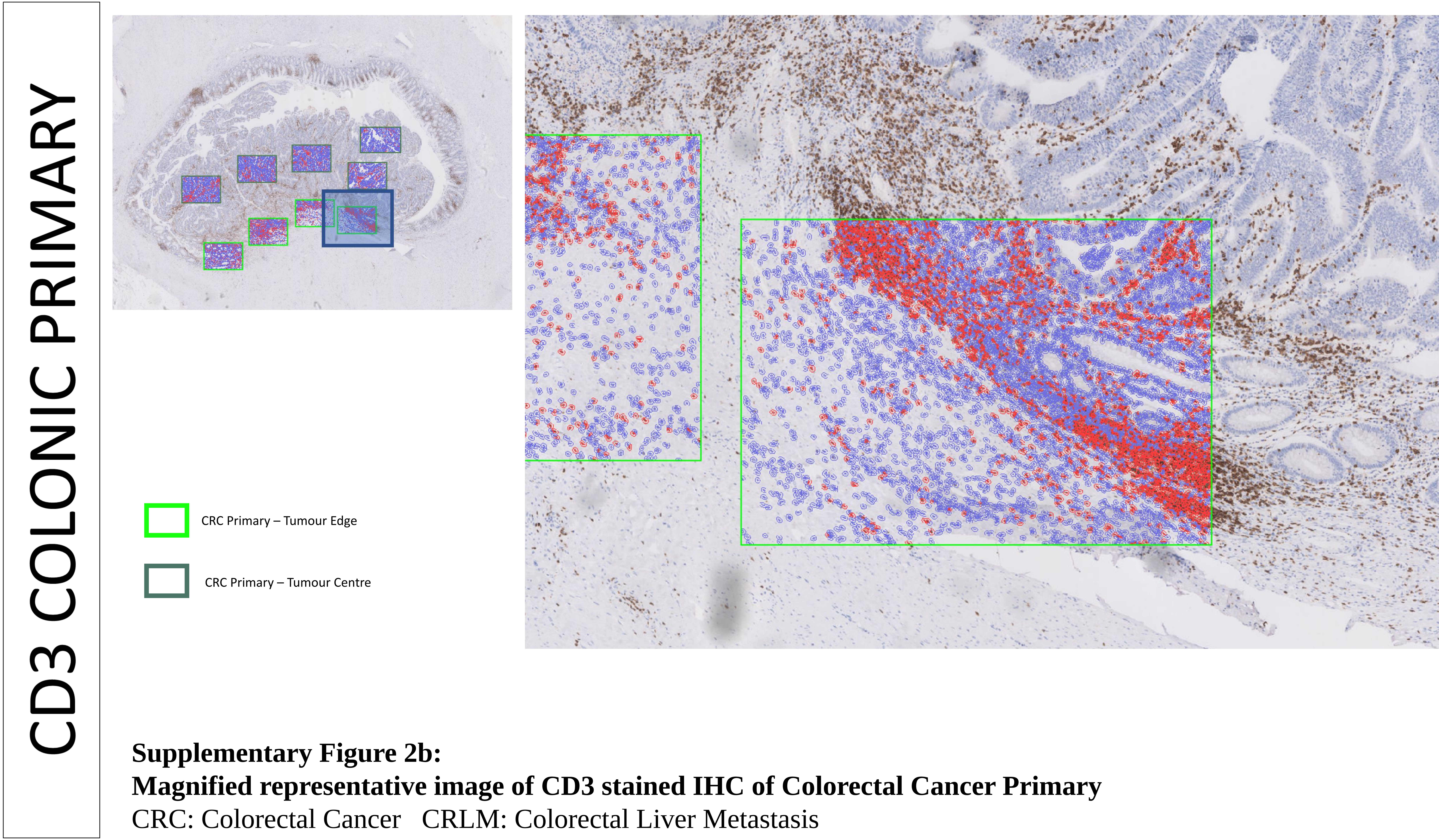

Supplementary Figure 2b:
Magnified representative image of CD3 stained IHC of Colorectal Cancer Primary
CRC: Colorectal Cancer   CRLM: Colorectal Liver Metastasis

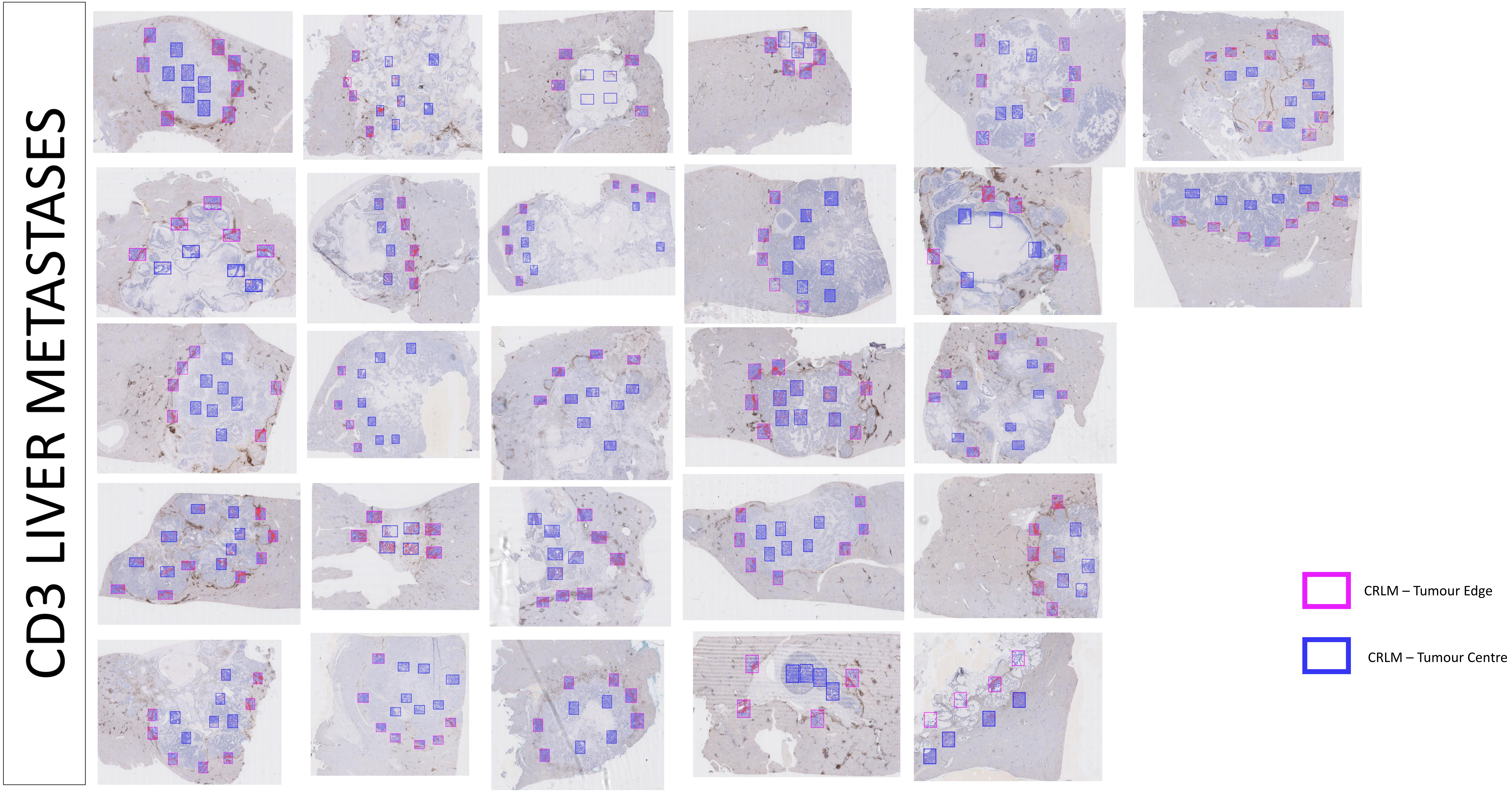

Supplementary Figure 2c:

Representative images of CD3 stained IHC of Colorectal Cancer Liver Metastases

CRC: Colorectal Cancer   CRLM: Colorectal Liver Metastasis

CD3 LIVER METASTASES

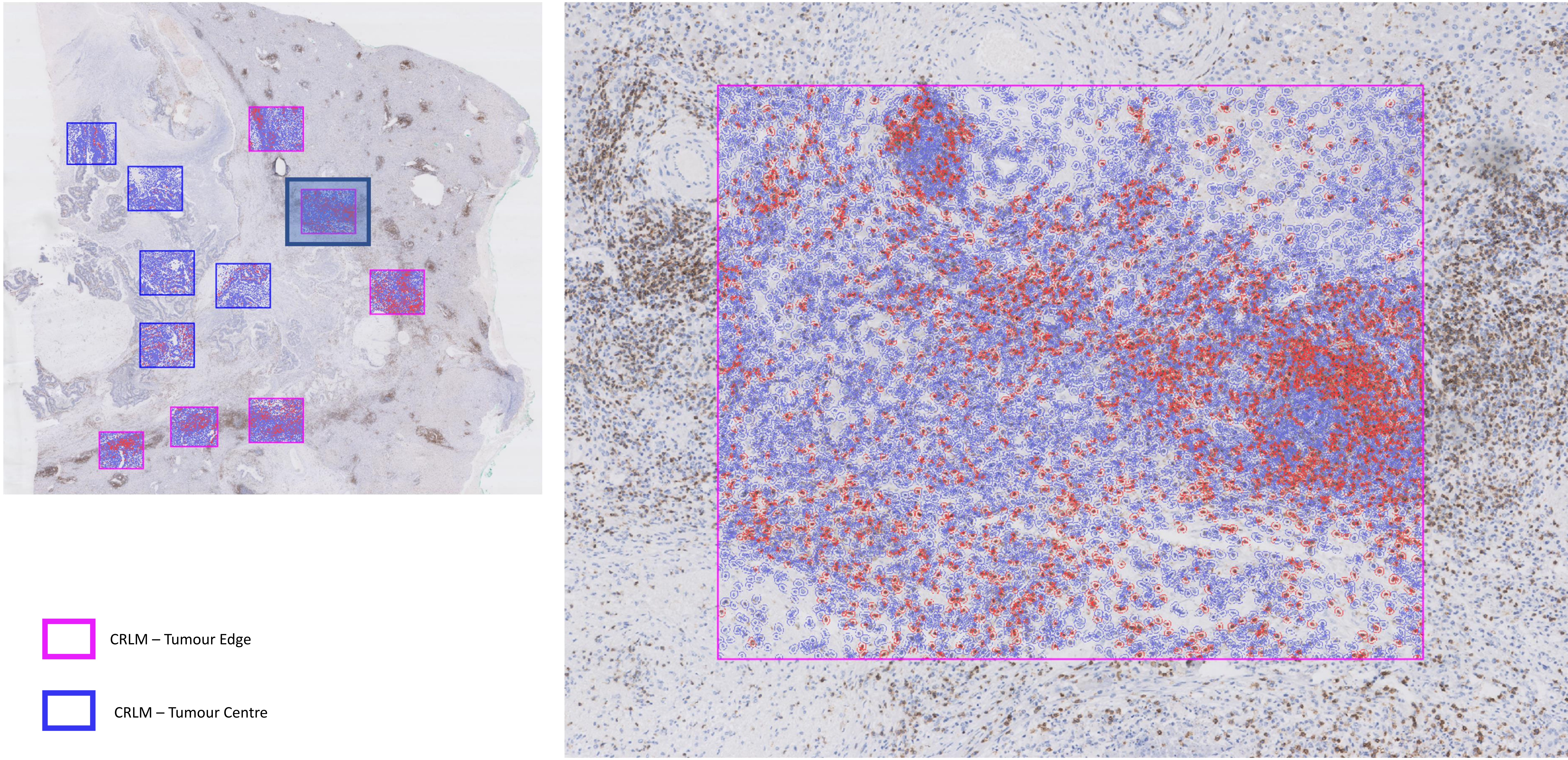

Supplementary Figure 2d:
Magnified representative images of CD3 stained IHC of Colorectal Cancer Liver Metastases
CRC: Colorectal Cancer   CRLM: Colorectal Liver Metastasis

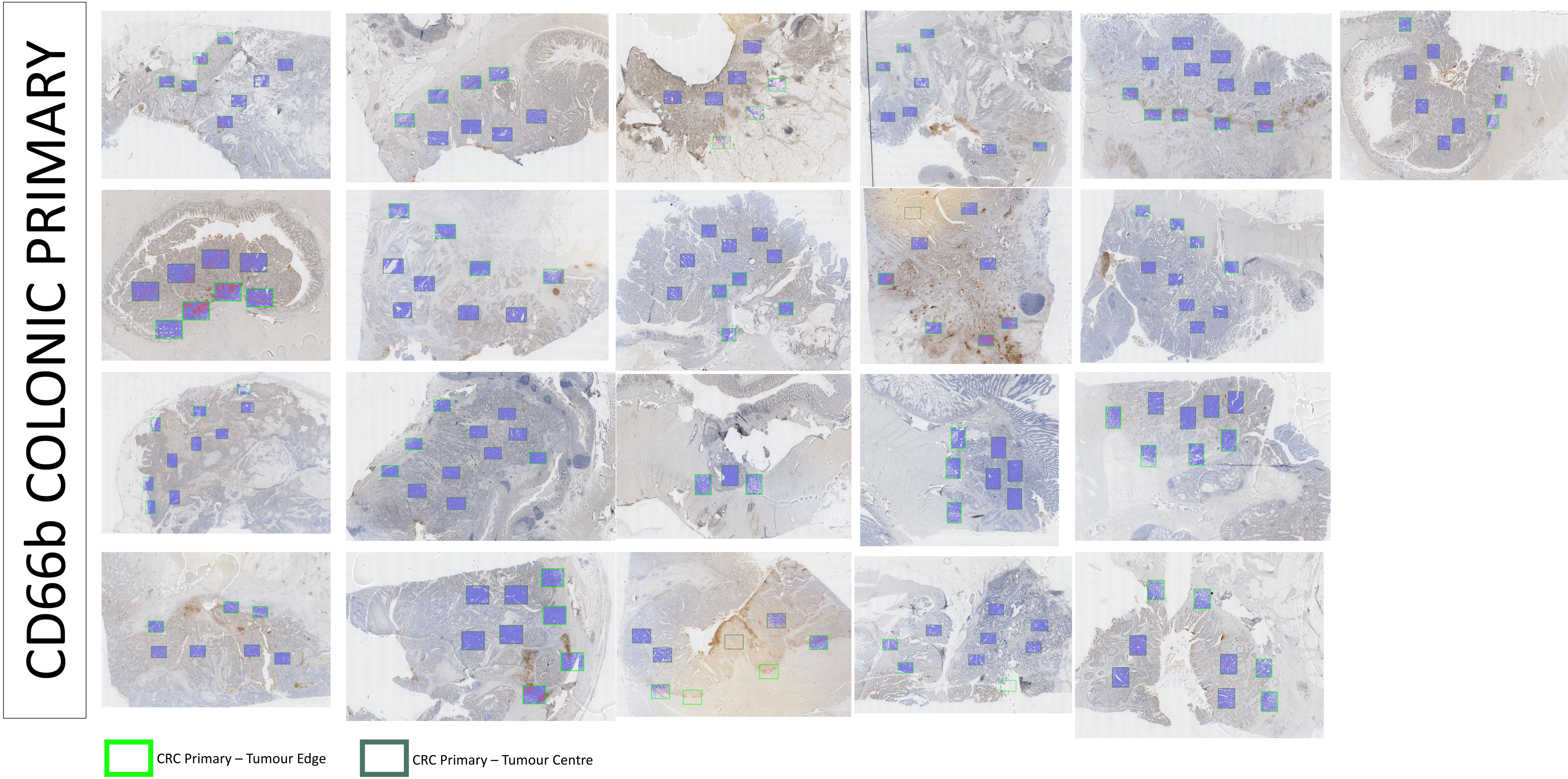

Supplementary Figure 2e:
Representative images of CD66b stained IHC of Colorectal Cancer PrimaryCRC: Colorectal Cancer
CRLM: Colorectal Liver Metastasis

CD66b COLONIC PRIMARY

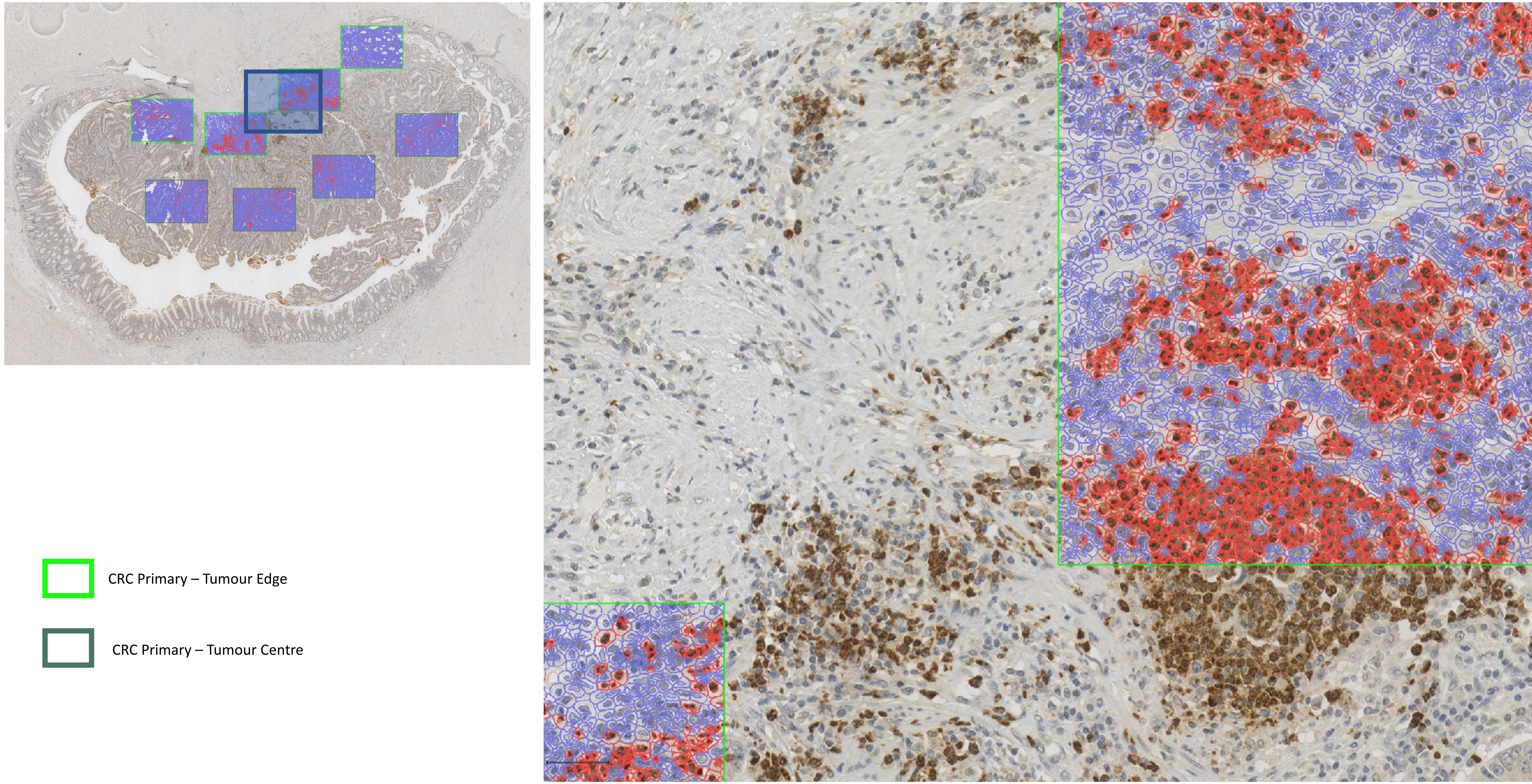

Supplementary Figure 2f:
Magnified representative images of CD66b stained IHC of Colorectal Cancer Primary
CRC: Colorectal Cancer   CRLM: Colorectal Liver Metastasis

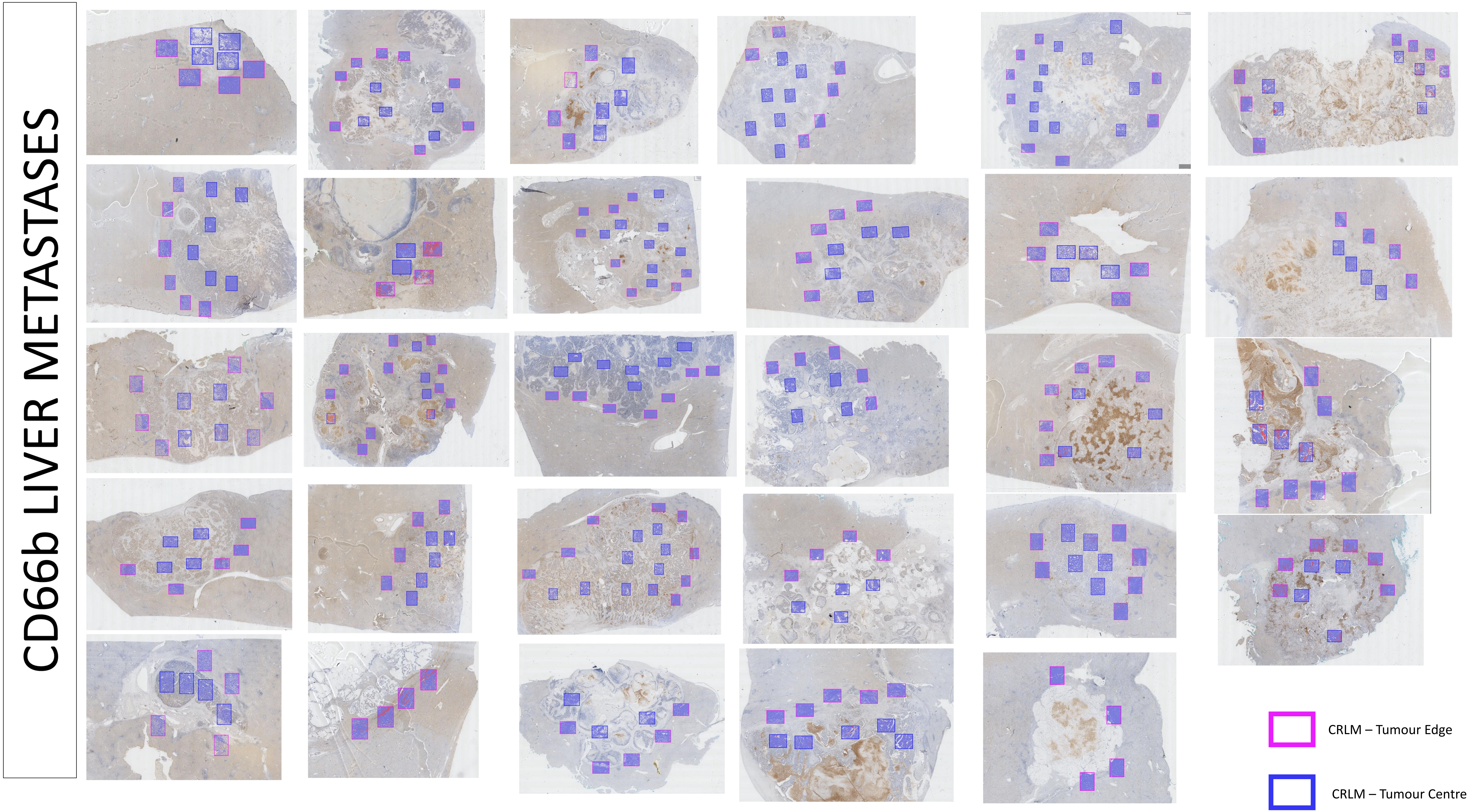

Supplementary Figure 2g: Representative images of CD66b stained IHC of Colorectal Cancer Liver Metastases

CRC: Colorectal Cancer   CRLM: Colorectal Liver Metastasis

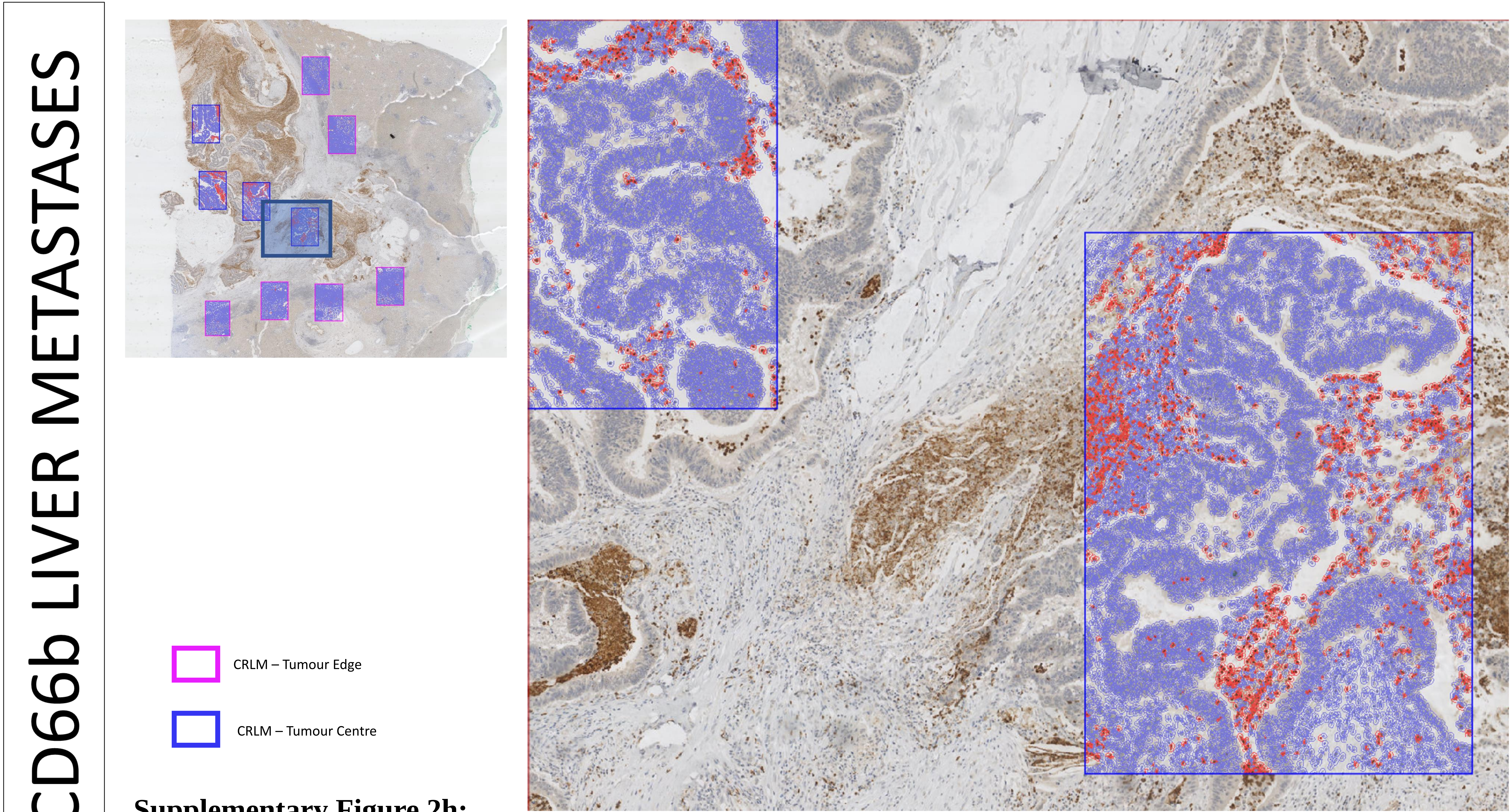

Supplementary Figure 2h:
Magnified representative images of CD66b stained IHC of Colorectal Cancer Liver Metastases
CRC: Colorectal Cancer   CRLM: Colorectal Liver Metastasis

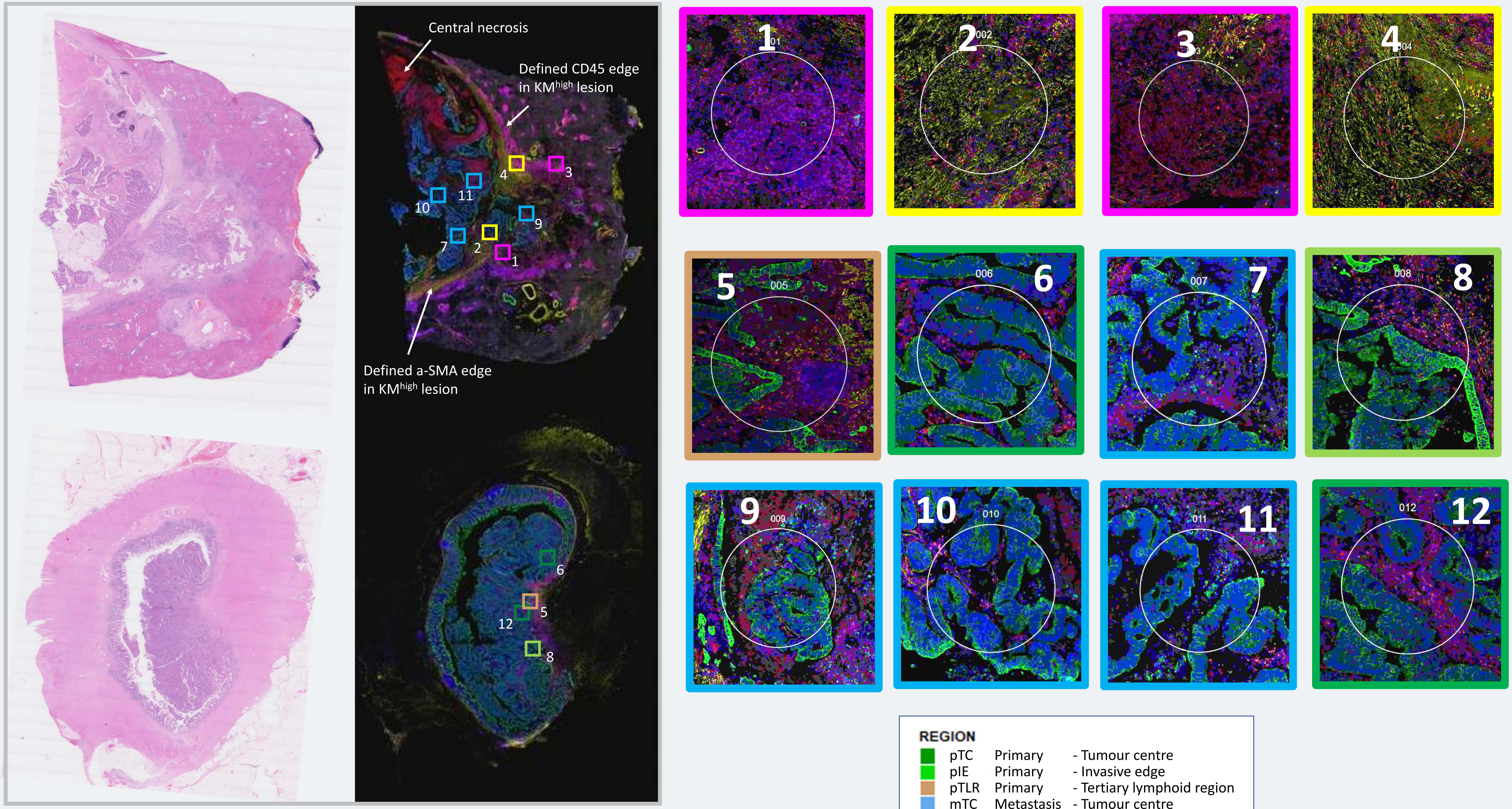

REGION

pTC	Primary	- Tumour centre
pIE	Primary	- Invasive edge
pTLR	Primary	- Tertiary lymphoid region
mTC	Metastasis	- Tumour centre
mIE	Metastasis	- Invasive edge (immune)
mSE	Metastasis	- Invasive edge (stromal)
mTLR	Metastasis	- Tertiary lymphoid region

Supplemental Figure 3 Patient A: KM^{high}, KRAS wildtype type

Representative H+E and mIF stained sample with annotated regions colour coded alongside magnified image of regions, 4 primary and 8 metastases, from patient A from GeoMx experiment.

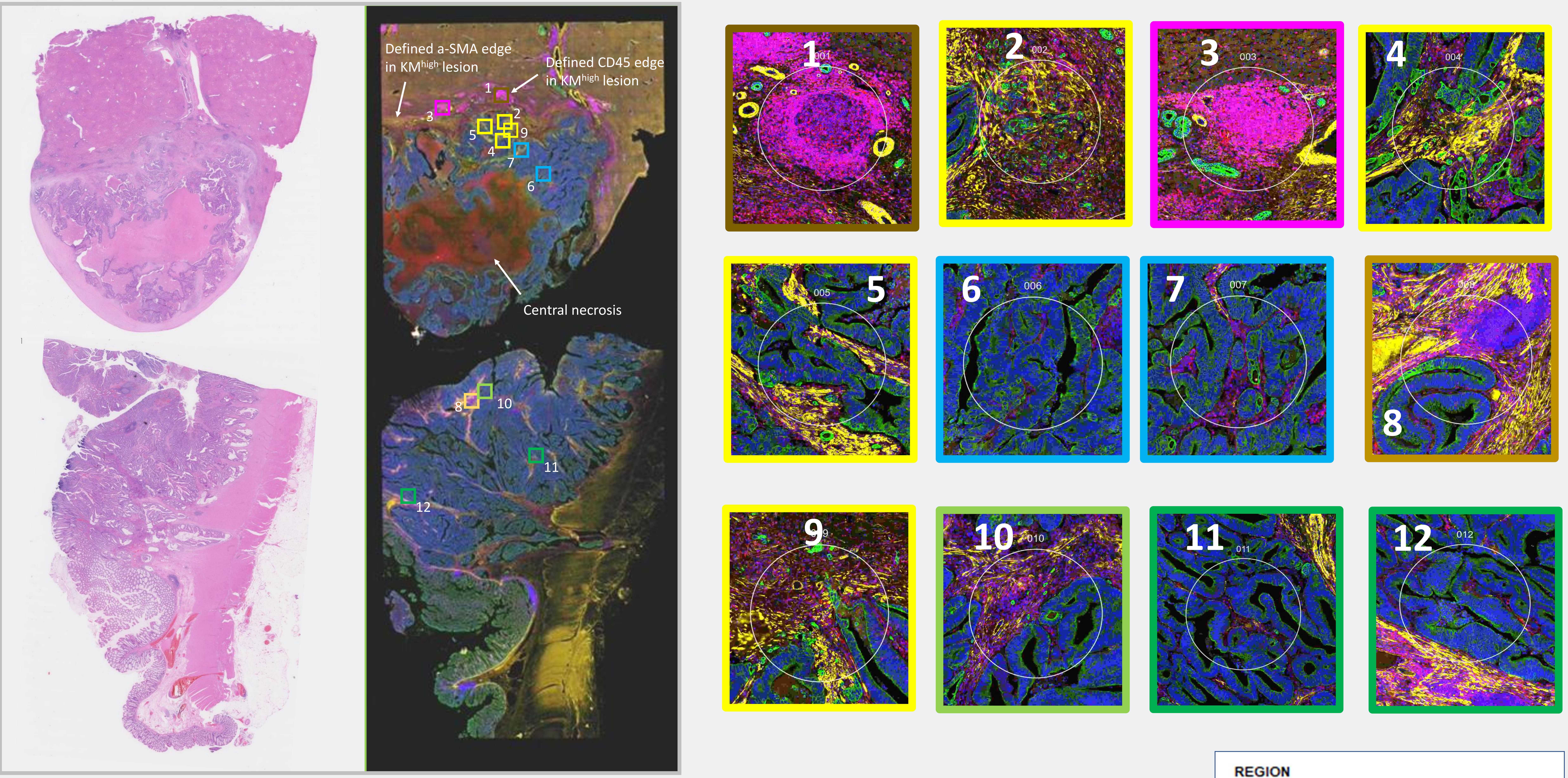

Supplemental Figure 4 **Patient B: KM^{high} immune, KRAS mutation**

Representative H+E and mIF stained sample with annotated regions colour coded alongside magnified image of regions, 4 primary and 8 metastases, from patient B from GeoMx experiment

REGION

pTC	Primary	- Tumour centre
pIE	Primary	- Invasive edge
pTLR	Primary	- Tertiary lymphoid region
mTC	Metastasis	- Tumour centre
mIE	Metastasis	- Invasive edge (immune)
mSE	Metastasis	- Invasive edge (stromal)
mTLR	Metastasis	- Tertiary lymphoid region

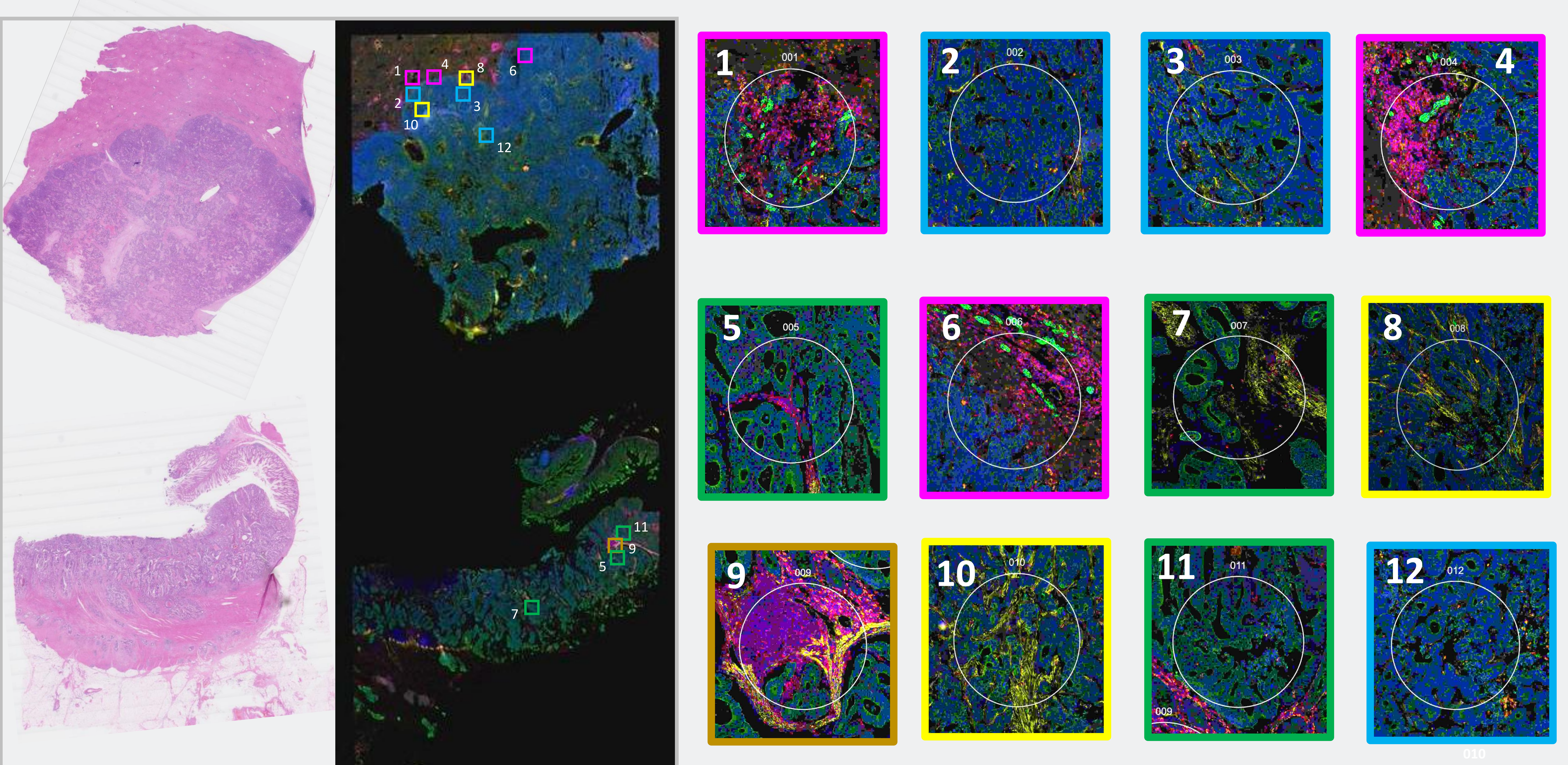

Supplemental Figure 5 **Patient C: KM^{low} immune, KRAS mutation**

Representative H+E and mIF stained sample with annotated regions colour coded alongside magnified image of regions, 4 primary and 8 metastases, from patient C from GeoMx experiment

REGION

pTC	Primary	- Tumour centre
pIE	Primary	- Invasive edge
pTLR	Primary	- Tertiary lymphoid region
mTC	Metastasis	- Tumour centre
mIE	Metastasis	- Invasive edge (immune)
mSE	Metastasis	- Invasive edge (stromal)
mTLR	Metastasis	- Tertiary lymphoid region

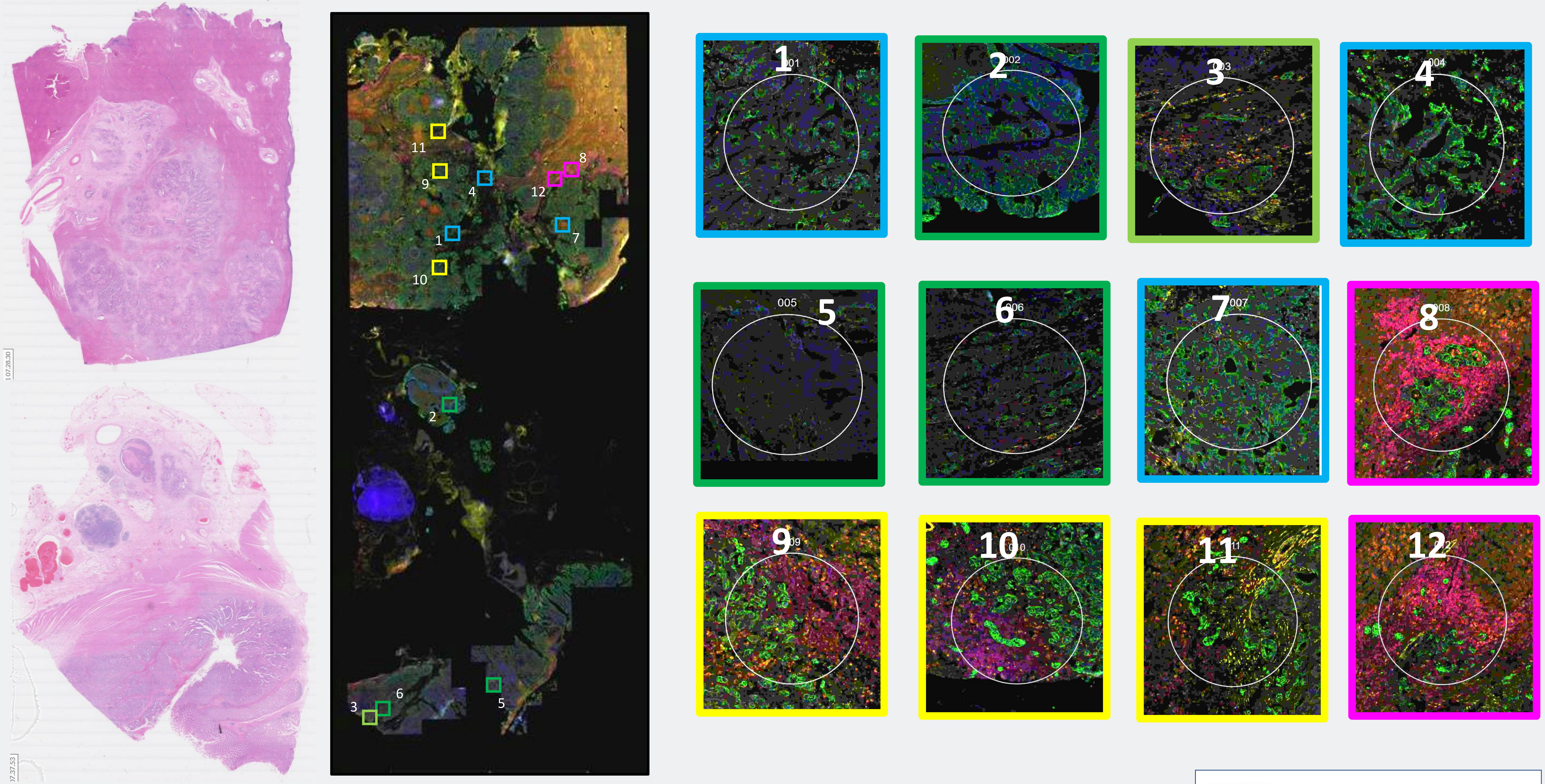

REGION

pTC	Primary	- Tumour centre
pIE	Primary	- Invasive edge
pTLR	Primary	- Tertiary lymphoid region
mTC	Metastasis	- Tumour centre
mIE	Metastasis	- Invasive edge (immune)
mSE	Metastasis	- Invasive edge (stromal)
mTLR	Metastasis	- Tertiary lymphoid region

Supplemental Figure 6 Patient D: KM^{low} immune, KRAS wildtype, BRAF mutant

Representative H+E and mIF stained sample with annotated regions colour coded alongside magnified image of regions, 4 primary and 8 metastases, from patient D from GeoMx experiment

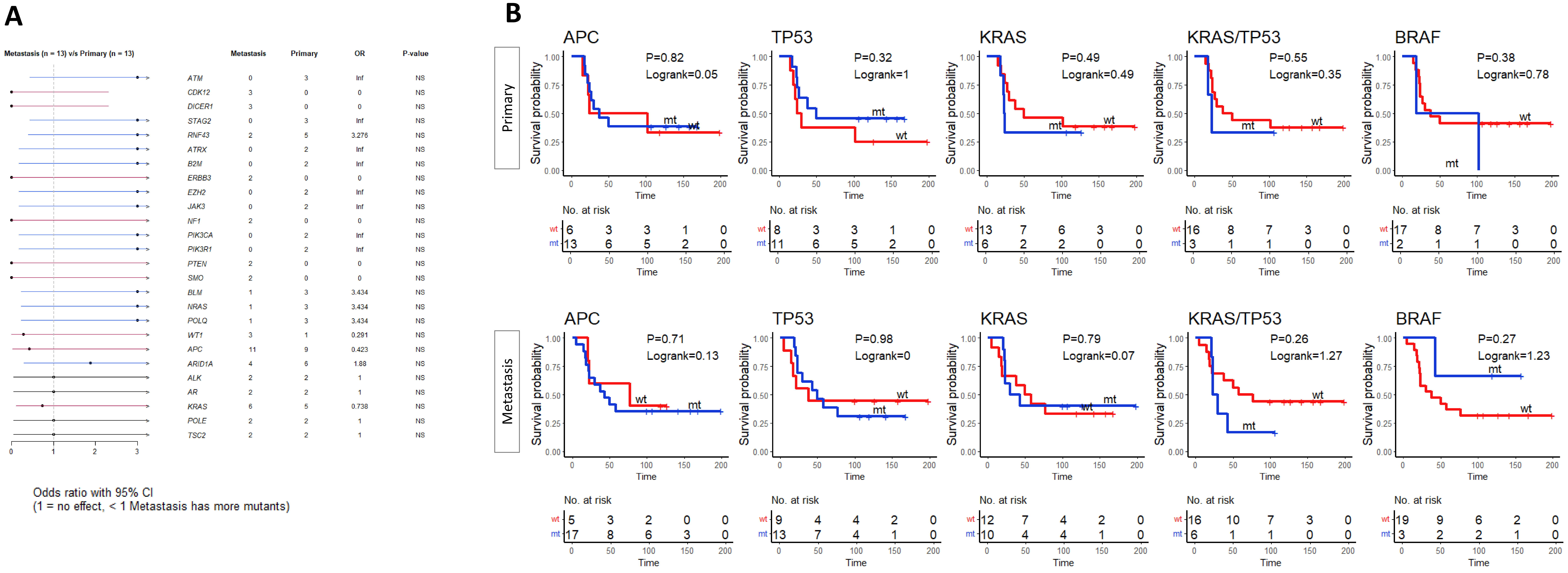

Supplemental Figure 7

A: Forrest plot demonstrating mutational discordance between primary and secondary (non-matched lesions)

B: Kaplan Meier survival plots for cancer specific survival by mutational groups with Log-rank test and P-value displayed.

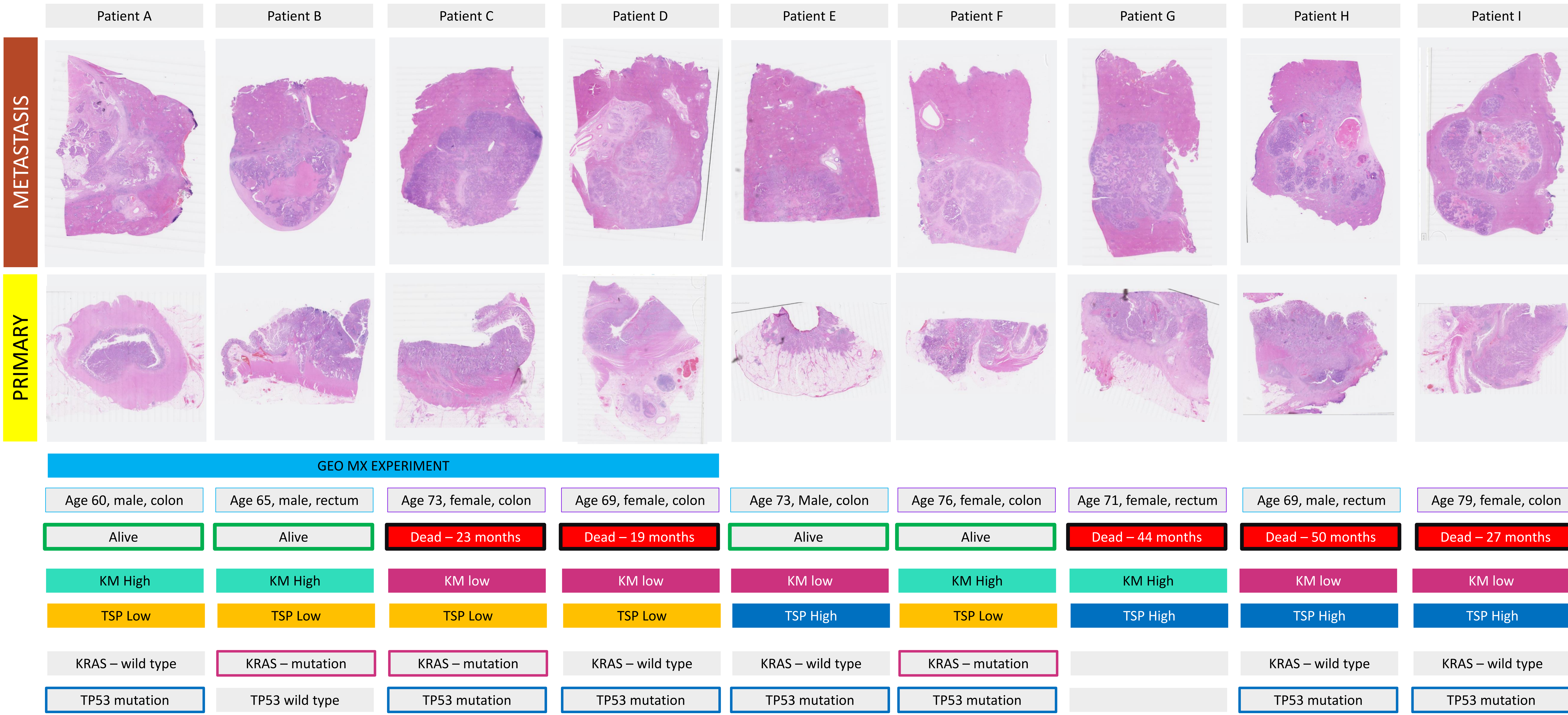

Supplemental Figure 8: Representative H+E and clinicopathological, mutational and survival details of patients (n=9) undergoing Bulk transcriptomic analysis on the nCounter platform

9 patients from the cohort (patients A-D in Supplemental Figures 2-4) had bulk transcriptomic analysis on the ncounter platform using the Pancancer IO360 (770 genes) panel with clinicopathological, morphological and mutational characteristics as demonstrated. Representative H+E from the primary and metastatic lesions from the patients are demonstrated. 4 of the patients were selected for analysis on the GeoMx Digital Spatial Profiler. A selection of KM and TSP score were selected for comparison.

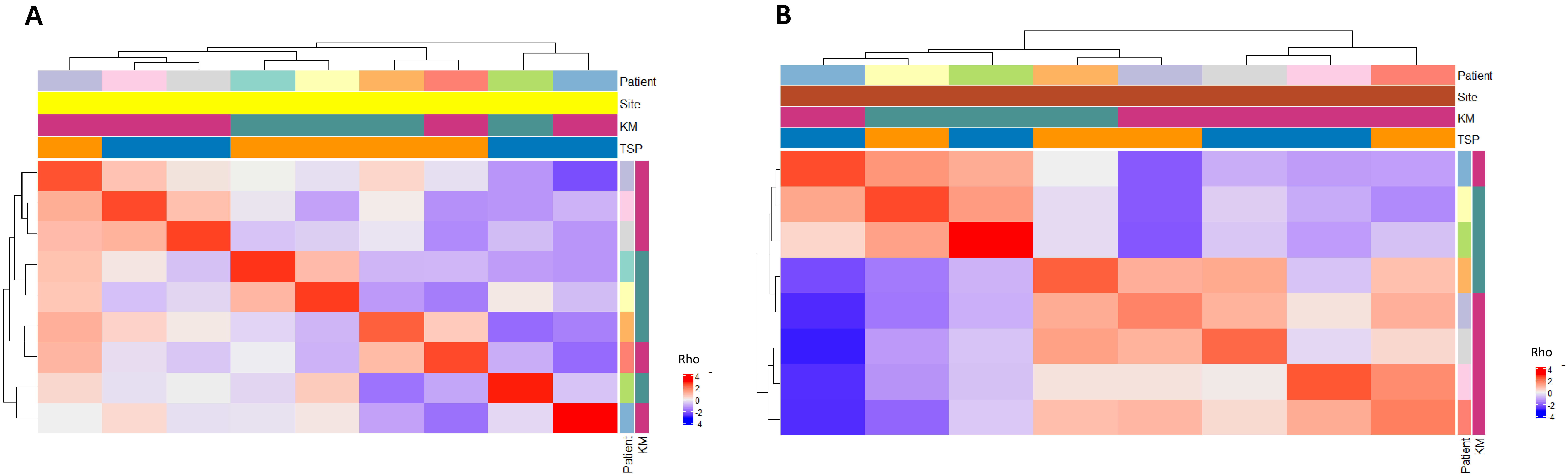

Supplemental Figure 9

A: Unsupervised analysis using gene expression correlation matrix for Primary lesions only. Patient, site, KM grade, tumor stromal percentage (TSP) are depicted by key. Spearman correlation of all expressed genes performed between each sample sequenced and plotted on the heatmap. k-means clustering of heatmap to demonstrate correlated samples. Red represents strong correlation. Blue represents negative correlation

B: Unsupervised analysis using gene expression correlation matrix for Metastatic lesions. Patient, site, KM grade, tumor stromal percentage (TSP) are depicted by key. Spearman correlation of all expressed genes performed between each sample sequenced and plotted on the heatmap. k-means clustering of heatmap to demonstrate correlated samples. Red represents strong correlation. Blue represents negative correlation

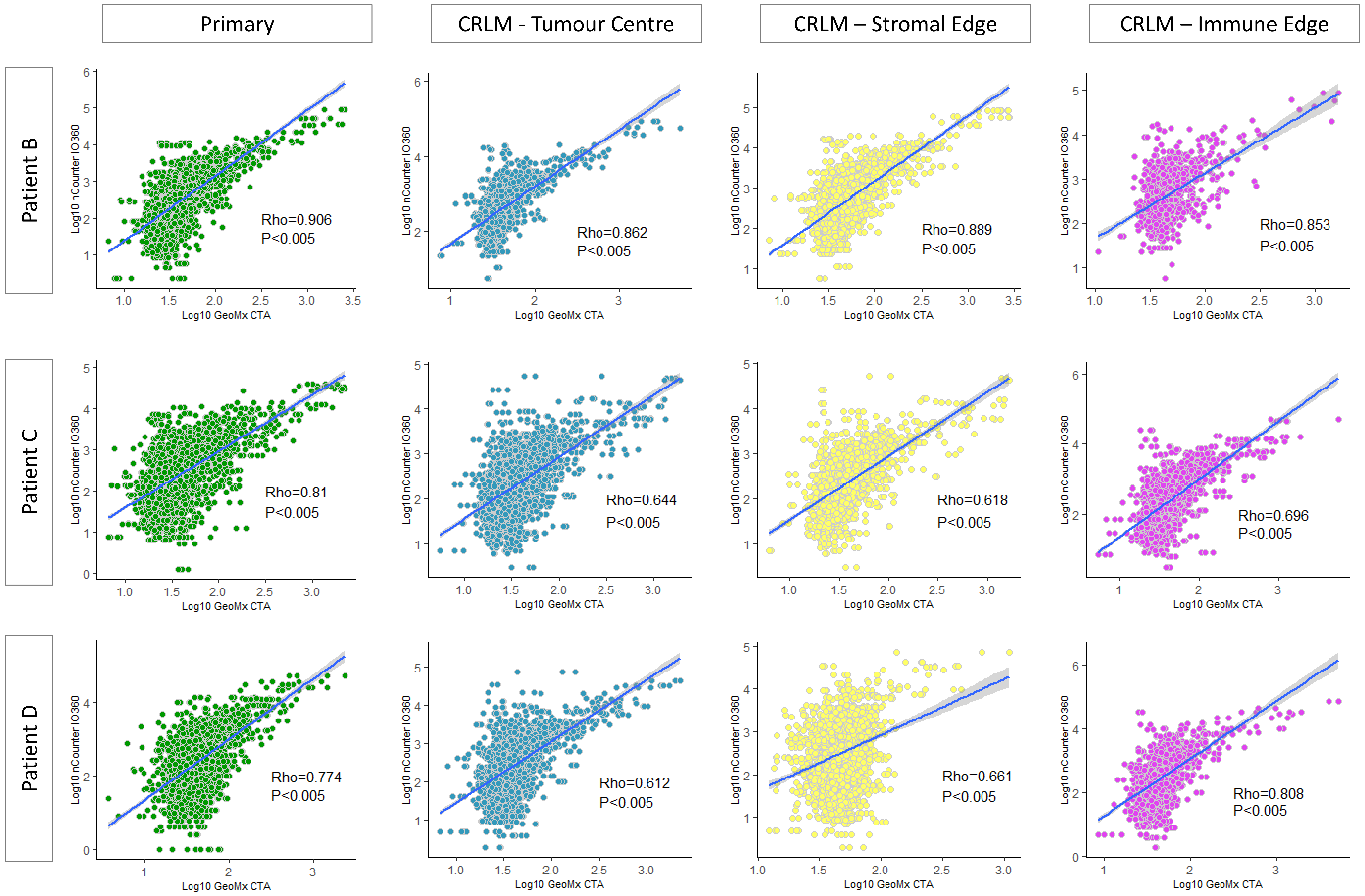

Supplemental Figure 10
Comparison of nCounter IO360 and GeoMx CTA Gene Expression in Matched Samples

Each dot represents log10 expression of gene on nCounter IO360 (y-axis) and GeoMx (x-axis) organised by region and patient.

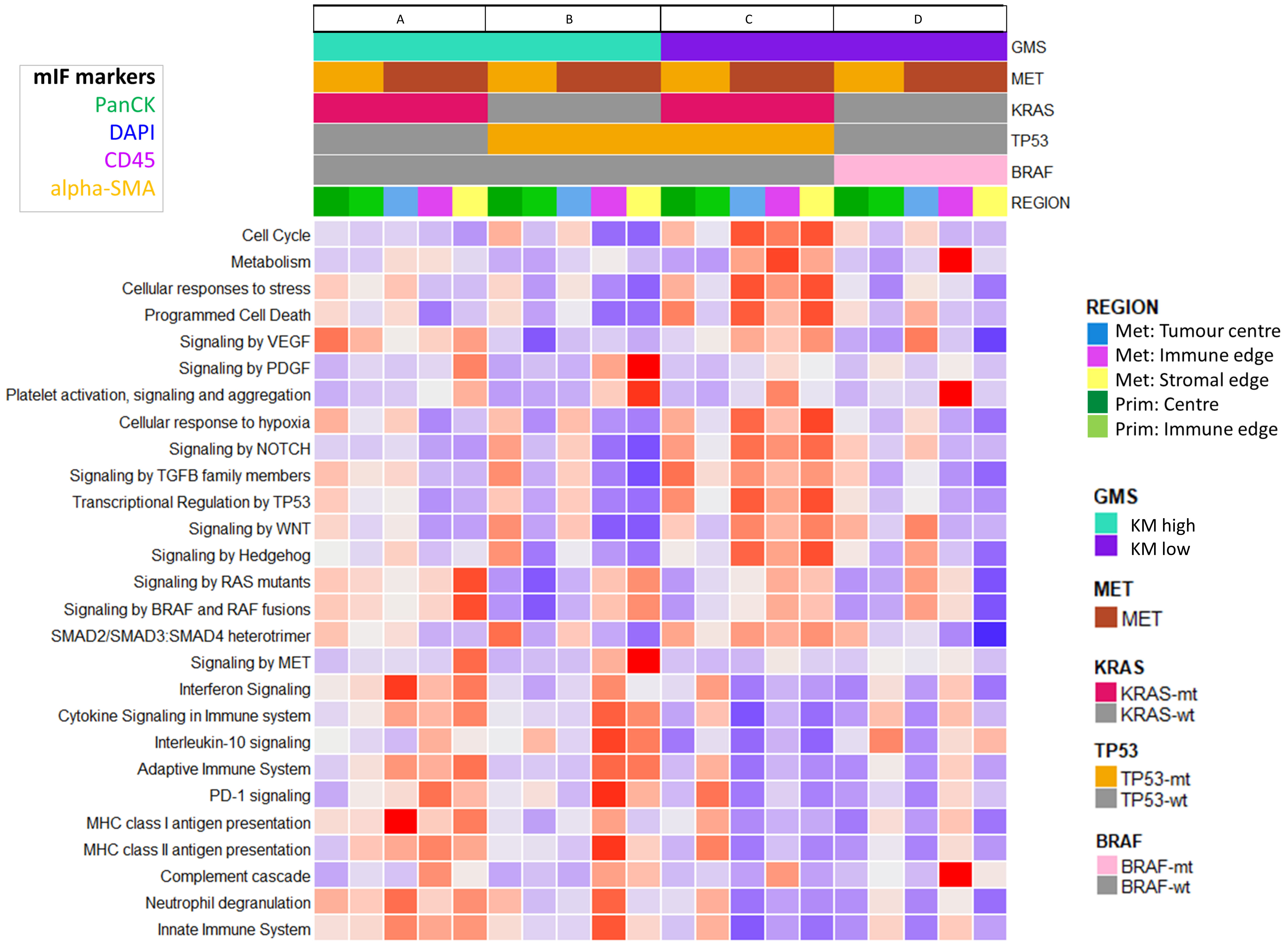

Supplemental Figure 11

Spatially Resolved Transcriptomic Analysis using REACTOME curated gene sets

Unclustered heatmap demonstrating summarised mean gene expression for each selected REACTOME pathway with annotations as shown. Ordered by patient, lesion (primary, metastasis) and annotated topographical region.

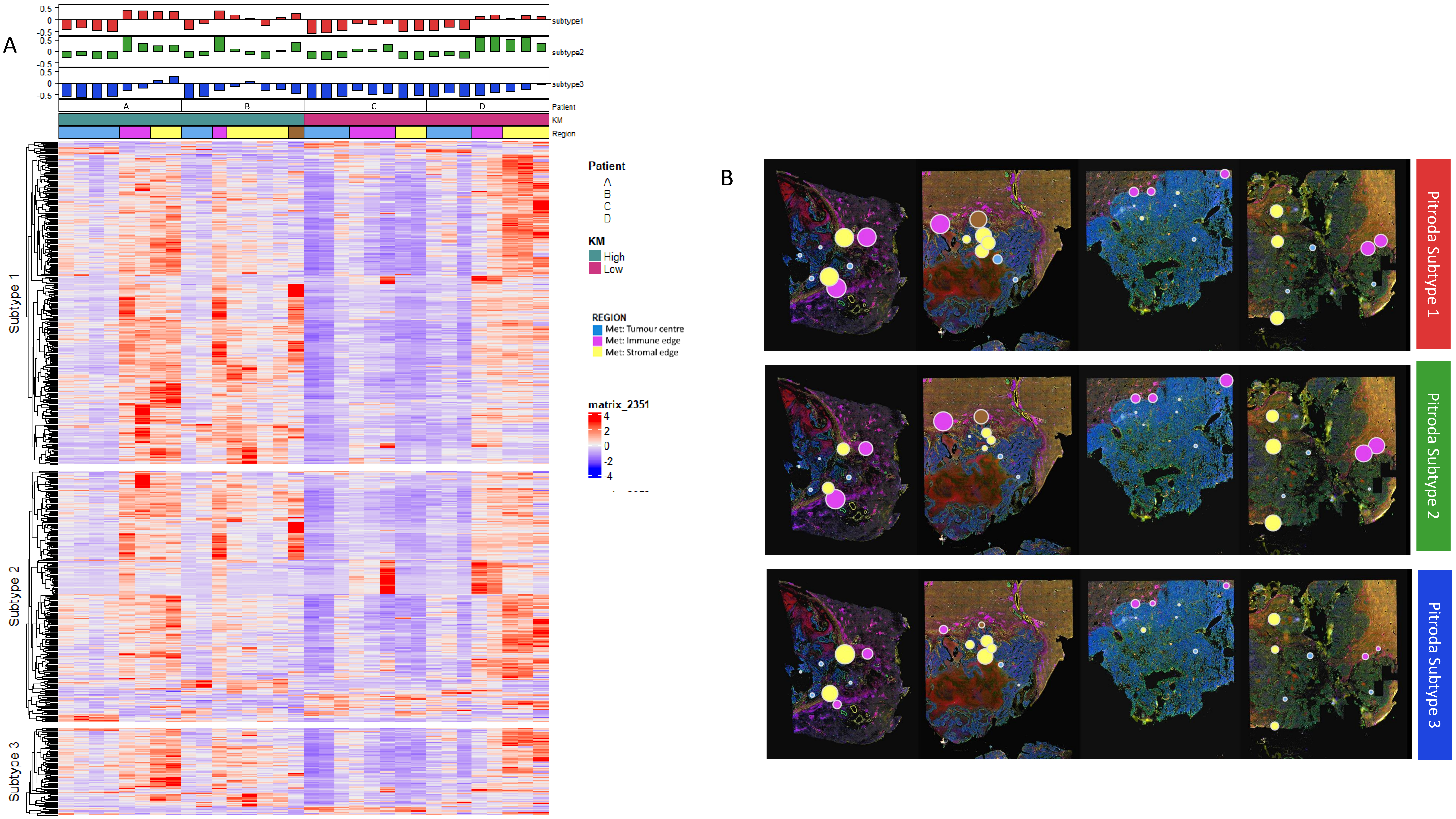

Supplementary Figure 12

A: Pitroda et al gene sets applied across spatial transcriptomic data set. ssGSEA enrichment score at top of heat map. Heat map comparing expression across CRLM and region. Overlap between CTA and Pitroda geneset, Subtype 1: (348 of 1506 genes) Subtype 2: (270 of 1259 genes) Subtype 3: (94 of 603 genes)

B: Geneset Enrichment score for each Pitroda subtype demonstrated by radius of region and topographic location on tumour. Annotated region demonstrated by color

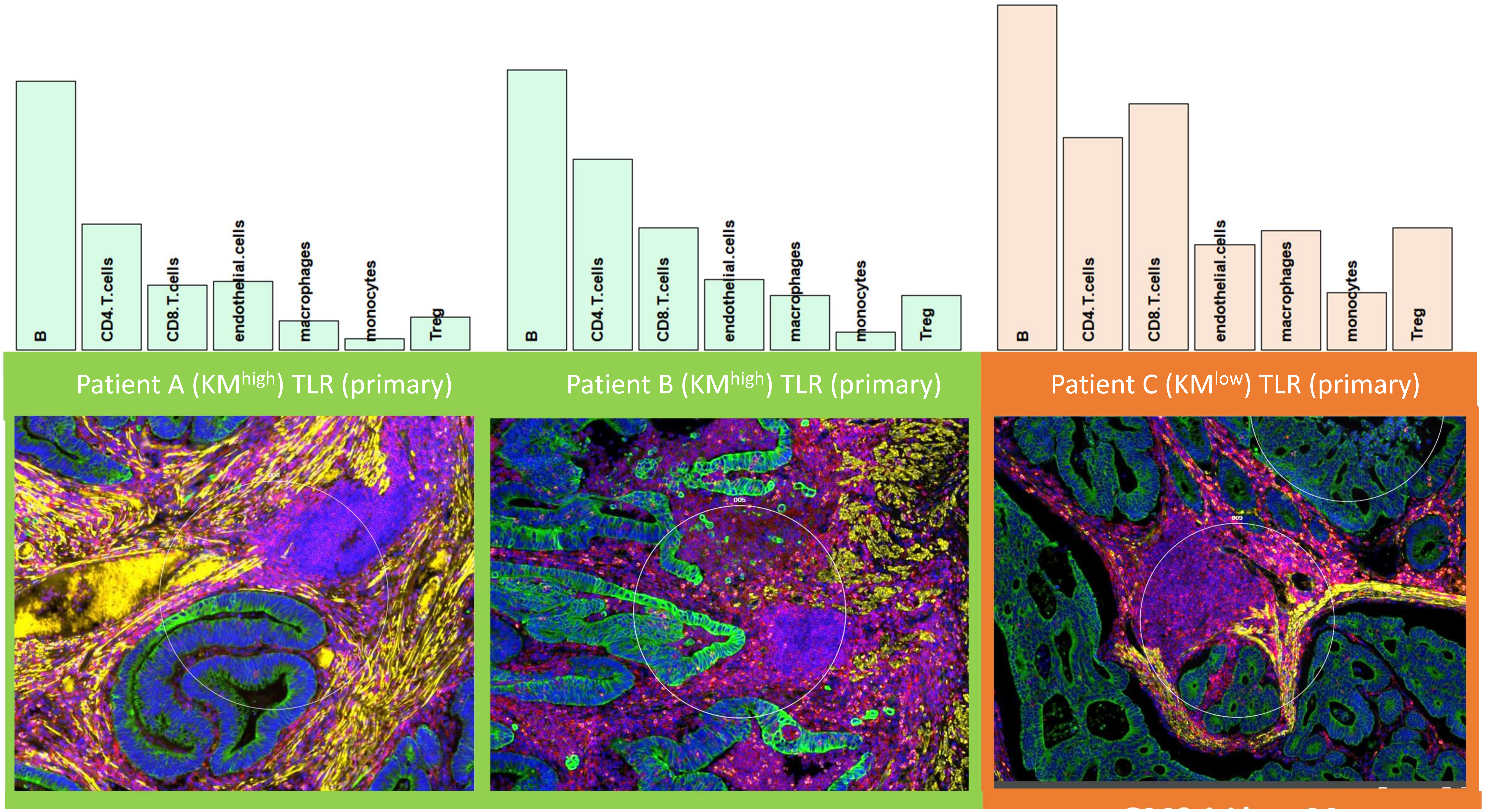

Supplementary Figure 13: Immune Cell Spatial Deconvolution of Tertiary Lymphoid Regions

The immune cell populations are plotted for Tertiary Lymphoid Regions in Lesions A,B (KM-high) (Patient A, image 5, Supplemental Figure 3 and Patient B, image 1, Supplemental Figure 4) and Lesion C (KM-low) (Patient C, image 9, Supplemental Figure 5).
