# Supplementary material for: Spatially Resolved Transcriptomics Deconvolutes Prognostic Histological Subgroups in Patients with Colorectal Cancer and Synchronous Liver Metastases
Source: Cancer Res. 2023 Apr 14;83(8):1329–44. doi: 10.1158/0008-5472.CAN-22-2794 (PMC10102851; doi:10.1158/0008-5472.CAN-22-2794)
Supplement: Supplementary Methods — Supplementary details regarding methods and materials [file can-22-2794_supplementary_methods_suppsmm.docx]

## Supplemental Methods

#### Immunohistochemistry

Sections were dewaxed in histoclear and rehydrated through a series of graded alcohols. Antigen retrieval was performed using TRIS-EDTA buffer pH8 for CD3, TRIS-EDTA buffer pH9 for CD66b. Sections were heated in respective buffer for 5 mins under pressure and then cooled for 30 mins. Endogenous peroxidase activity was blocked by adding slides to 3% hydrogen peroxide for 20 mins. Sections were washed in running water and then blocked for 1 hour at room temperature using 5% goat serum. Sections were incubated overnight at 4°C in primary antibody diluted in antibody diluent (Dako) at the following concentrations: CD3 (Sigma) 1:4000, CD66b (Novus) 1:4000. After overnight incubation, sections were washed in tris-buffered saline (TBS) and ImmPRESS reagent was applied for 30 mins at room temperature. Sections were washed in TBS and DAB substrate was added for 5-10 mins before washing in running water. Counterstaining was performed using haematoxylin for 5 minutes, rinsed in water, 1-2 seconds in acid-alcohol, rinsed in water, 45 seconds in Scotts tap water substitute. Sections were dehydrated through a series of graded alcohols, passed through histoclear and coverslips were mounted using distrene plasticizer xylene.

#### Nanostring nCounter PanCancer IO360 Bulk Transcriptomic Platform

##### RNA extraction from FFPE tissue

RNA was extracted from the samples using AllPrep DNA/RNA FFPE Kit (Qiagen) according to manufacturer’s protocol, using xylene for deparaffinisation. All RNA samples were treated on-column with DNAse I and were eluted in 20ul RNAse-free H_2_O. RNA quantity was assessed using RNA BR assay and the Qubit^®^ 2.0 fluorometer (Invitrogen, Life Technologies). RNA integrity (RIN) values were determined using Agilent 2100 Bioanalyzer (Agilent Technologies) (maximum RIN = 2.1).

##### Gene Expression Profiling using NanoString Immune Oncology (IO 360) Panel

Total RNA (Average concentration = 35ng/µl) was used for gene expression analysis using the commercially available IO360 panel (nCounter, NanoString) (770 genes). Each 5µl RNA sample was analysed in sets of 11. Panel standards were used in each assay to allow for differences in technical assay performance across runs. Samples and panel standards were spiked with positive control probes ranging from 128fM to 0.125fM in a four-fold dilution series. Positive controls were used to assess the linearity of the assays and used for normalisation. Additionally, eight negative control probes were used for which no RNA target was present. RNA samples were incubated for 16 hours at 65°C in hybridisation buffer containing IO360 CodeSet, including reporter and capture probes in addition to target RNA forming a tripartite hybridisation complex. Hybridized samples were processed using the nCounter Prep Station (High Sensitivity Protocol), in accordance with IO360 panel guidelines by Nanostring. Data acquisition was performed by using the Nanostring’s Digital Analyser (FOV, 555).

##### Gene expression analysis

Raw gene expression count data was normalized in NanoString nSolver 4.0 using 6 positive controls and 8 negative controls to account for background noise and sample variation across several runs performed on the nCounter platform. Background threshold was manually calculated using Mean (negative controls) ±2 standard deviations of negative control probes to remove lowly expressed targets prior to gene expression analysis. Then, the nSolver 4.0 software enabled data normalisation to be performed using the GeNorm Algorithm with integrated housekeeping gene probes.

#### Nanostring GeoMx Digital Spatial Profiling

##### Slide preparation and hybridisation with UV-photocleavable CTA probes

The four matched 5μm-thick FFPE colon cancer and corresponding liver metastasis tissues were co-mounted on Superfrost glass slides (Thermofischer). The slides were baked for 30 mins at 60 °C. The tissues were dewaxed, hydrated and treated with 1μg/ml Proteinase K for 15 minutes. Next, heat-induced epitope retrieval (HIER) was performed on the Leica BOND Autostainer (ER2 for 100°C) for 20 minutes using a pH 9 antigen retrieval buffer (Leica BOND). The slides were immediately stored in 1x PBS. The tissues were then hybridized as per manufacturer’s protocol with a pre-designed panel of antibodies corresponding to 1,825 genes, known as the Cancer Transcriptome Atlas (CTA) (Nanostring). The CTA is a panel of 1825 genes designed to comprehensively characterize immune activity and tumor biology within tumor microenvironments (46). Each tissue was covered with 200μL of hybridization solution and a HybriSlip™ cover and incubated overnight at 37 °C for at least 16 hours. The slides were then dipped in 2x SSC-T and washed twice with a 1:1 ratio of 100% deionized formamide (Ambion) and 4x SSC at 37°C for 25 minutes each. The slides were blocked with Buffer W (Nanostring) before the addition of fluorescently-labelled morphology markers on the tissue to highlight the tissue’s architecture. The GeoMx DSP hosts four channels (FITC/525nm, Cy3/568nm, Texas Red/615nm and Cy5/666nm) for the detection of up to four customisable morphology markers for each tissue (20). One channel is reserved for the nuclear stain (DAPI), which left three free channels. Consistent with the aim of this project, the additional morphology markers were Pan-Cytokeratin (PanCK) to stain the cytokeratin in the epithelium, cluster of differentiation, CD45, for the immune cell populations and α-SMA to identify fibrogenetic collagenous architecture in liver tissue. The slides were then stored at 4°C in SSC before being loaded on the GeoMx DSP instrument for region selection and collection.

##### Probe collection and Library Preparation

Once region selection was complete, collection was initiated using the DSP workstation whereby photo-cleavable oligonucleotide probes in the user-defined regions were exposed to UV-light to cleave the UV-sensitive probes which releases the probe-specific DSP barcodes which are aspirated from selected regions and dispensed. Following collection, the DSP plate containing the probes was dried and rehydrated with DEPC-treated water before each sample was added to the corresponding well of a new 96-well PCR plate containing the GeoMx Seq Code primers and the PCR Master Mix. A thermocycler was user to incubate the PCR plate using manufacturer settings (Supplemental table 1). The products of the PCR reaction were then centrifuged and pooled prior to purification which was performed using AMPure XP system. Once purified, the library was resuspended in Elution Buffer (10mM Tris-HCl with 0.05% Tween-20, pH 8.0 prior to QC using an Agilent Bioanalyser. Having met Nanostring’s recommendation, the experiment proceeded to NGS.

##### Sequencing

The purified library was loaded onto a flow cell and placed on the Illumina NextSeq 550, where the RNA fragments were amplified in a process called cluster generation. Then, during sequencing by synthesis (SBS), fluorescently-labelled complementary nucleotides bind and read the RNA sequence in a two-way fashion – first forward and then reverse – through a method known as paired-end sequencing generating. The GeoMx NGS pipeline hosted in the Illumina BaseSpace platform was used to convert sequenced FASTQ files into DCC files which can be uploaded onto the GeoMx DSP analysis suite. Once loaded, they undergo quality control, filtering, Q3 normalisation and background correction. The counts were then downloaded from the GeoMx instrument and loaded in to RStudio (v1.2.1335) using R build version 4.1.1 for analysis.
